# Interpregnancy interval and adverse pregnancy outcomes among pregnancies following miscarriages or induced abortions in Norway (2008–2016): A cohort study

**DOI:** 10.1371/journal.pmed.1004129

**Published:** 2022-11-22

**Authors:** Gizachew A. Tessema, Siri E. Håberg, Gavin Pereira, Annette K. Regan, Jennifer Dunne, Maria C. Magnus

**Affiliations:** 1 Curtin School of Population Health, Curtin University, Perth, Western Australia, Australia; 2 School of Public Health, University of Adelaide, Adelaide, South Australia, Australia; 3 Centre for Fertility and Health, Norwegian Institute of Public Health, Oslo, Norway; 4 School of Nursing and Health Professions, University of San Francisco, Orange, California, United States of America; 5 Fielding School of Public Health, University of California Los Angeles, Los Angeles, California, United States of America; Cambridge University, UNITED KINGDOM

## Abstract

**Background:**

The World Health Organization recommends to wait at least 6 months after miscarriage and induced abortion before becoming pregnant again to avoid complications in the next pregnancy, although the evidence-based underlying this recommendation is scarce. We aimed to investigate the risk of adverse pregnancy outcomes—preterm birth (PTB), spontaneous PTB, small for gestational age (SGA) birth, large for gestational age (LGA) birth, preeclampsia, and gestational diabetes mellitus (GDM)—by interpregnancy interval (IPI) for births following a previous miscarriage or induced abortion.

**Methods and findings:**

We conducted a cohort study using a total of 49,058 births following a previous miscarriage and 23,707 births following a previous induced abortion in Norway between 2008 and 2016. We modeled the relationship between IPI and 6 adverse pregnancy outcomes separately for births after miscarriages and births after induced abortions. We used log-binomial regression to estimate unadjusted and adjusted relative risk (aRR) and 95% confidence intervals (CIs). In the adjusted model, we included maternal age, gravidity, and year of birth measured at the time of the index (after interval) births. In a sensitivity analysis, we further adjusted for smoking during pregnancy and prepregnancy body mass index. Compared to births with an IPI of 6 to 11 months after miscarriages (10.1%), there were lower risks of SGA births among births with an IPI of <3 months (8.6%) (aRR 0.85, 95% CI: 0.79, 0.92, *p* < 0.01) and 3 to 5 months (9.0%) (aRR 0.90, 95% CI: 0.83, 0.97, *p* = 0.01). An IPI of <3 months after a miscarriage (3.3%) was also associated with lower risk of GDM (aRR 0.84, 95% CI: 0.75, 0.96, *p* = 0.01) as compared to an IPI of 6 to 11 months (4.5%). For births following an induced abortion, an IPI <3 months (11.5%) was associated with a nonsignificant but increased risk of SGA (aRR 1.16, 95% CI: 0.99, 1.36, *p* = 0.07) as compared to an IPI of 6 to 11 months (10.0%), while the risk of LGA was lower among those with an IPI 3 to 5 months (8.0%) (aRR 0.84, 95% CI: 0.72, 0.98, *p* = 0.03) compared to an IPI of 6 to 11 months (9.4%). There was no observed association between adverse pregnancy outcomes with an IPI >12 months after either a miscarriage or induced abortion (*p* > 0.05), with the exception of an increased risk of GDM among women with an IPI of 12 to 17 months (5.8%) (aRR 1.20, 95% CI: 1.02, 1.40, *p* = 0.02), 18 to 23 months (6.2%) (aRR 1.24, 95% CI: 1.02, 1.50, *p* = 0.03), and ≥24 months (6.4%) (aRR 1.14, 95% CI: 0.97, 1.34, *p* = 0.10) compared to an IPI of 6 to 11 months (4.5%) after a miscarriage. Inherent to retrospective registry-based studies, we did not have information on potential confounders such as pregnancy intention and health-seeking bahaviour. Furthermore, we only had information on miscarriages that resulted in contact with the healthcare system.

**Conclusions:**

Our study suggests that conceiving within 3 months after a miscarriage or an induced abortion is not associated with increased risks of adverse pregnancy outcomes. In combination with previous research, these results suggest that women could attempt pregnancy soon after a previous miscarriage or induced abortion without increasing perinatal health risks.

## Abstract 2

In a Norwegian cohort study, Dr Gizachew Tessema and colleagues investigate the association between interpregnancy interval and adverse pregnancy outcomes, among pregnancies following miscarriages or induced abortions.

## Author summary

**Why was this study done?:**

- The World Health Organization recommends to wait at least 6 months after miscarriage and induced abortion before becoming pregnant again to avoid complications in the next pregnancy, although the evidence-based underlying this recommendation is scarce.
- The differences in pregnancy outcomes according to interpregnancy interval (IPI) after miscarriage as opposed to induced abortions remains unclear.

**What did the researchers do and find?:**

- Using data linkage from registry data in Norway, we explored the risks of adverse pregnancy outcomes for births after a miscarriage and after an induced abortion separately. While 3 out of 5 women with previous miscarriages conceive within 6 months, 1 out of 5 women with previous induced abortions conceive within 6 months.
- Our study suggests that conceiving within 6 months after a miscarriage or an induced abortion is not associated with increased risks of adverse pregnancy outcomes. The results are consistent for IPI as short as 3 months.
- There was no evidence of higher risks of adverse pregnancy outcomes among women with an IPI of greater than 12 months after a miscarriages or induced abortions, with the exception of a modest increased risk of gestational diabetes mellitus.

**What do these findings mean?:**

- Our results do not support current international recommendations to wait at least 6 months after a miscarriage or an induced abortion.
- In combination with previous research, our results are reassuring for families who attempt pregnancy soon after a miscarriage or induced abortion.
- These findings motivate a review of current international guidelines for birth spacing after a miscarriage or an induced abortion.

## Introduction

Miscarriage or the loss of the fetus before viability occurs in 12% to 15% of recognised pregnancies [1–3], and induced abortions occur in up to 15% of pregnancies in high-income countries [4]. Miscarriage causes significant psychological distress for couples [5], and induced abortions are performed for many different reasons including medical, financial, or social reasons [6,7]. The optimal interpregnancy interval (IPI)—the time between the end of one pregnancy and the start of the next—after pregnancy loss remains unclear. Based on a single study from Latin America, which reported that an IPI less than 6 months following miscarriages and induced abortions was associated with increased risk of adverse perinatal outcomes in the next pregnancy (odds ratio (OR) > 2.0 for preterm birth (PTB) and low birth weight (LBW)) [8], the World Health Organization (WHO) has recommended since 2007 that women wait at least 6 months before trying to become pregnant again after a miscarriage or induced abortion [9]. The study from Latin America did not distinguish between miscarriages and induced abortions, which is likely to have resulted in substantial heterogeneity in the underlying risk profile. Furthermore, when large cohort studies from Scotland (OR 0.89, 95% confidence interval (CI): 0.81, 0.98 for PTB; OR 0.84, 95% CI: 0.71, 0.89 for LBW; and OR 1.18, 95% CI: 0.82, 1.23 for preeclampsia) and California (OR 0.87, 95% CI: 0.81, 0.94 for PTB) subsequently evaluated this research question, they observed no increased risk of adverse pregnancy outcomes in births that followed a short IPI (<6 months) after a miscarriage [10,11]. However, an increased risk of adverse pregnancy outcomes in pregnancies that followed a short (<6 months) IPI after induced abortions was observed in Finland (OR 1.44, 95% CI: 1.10, 1.88 for PTB) [12]. Studies investigating risks of maternal complications after long IPI following a live birth indicated increased risks of gestational diabetes mellitus (GDM) in Canada (OR 1.66, 95% CI: 1.39, 2.00) and preeclampsia in Australia (OR 1.28, 95% CI: 1.17, 1.41) [13,14]. The differences in pregnancy outcomes according to IPI after miscarriage as opposed to induced abortions therefore remains unclear. Besides the available large cohort studies described above, other studies with smaller sample size (*n* < 1,100) were conducted to explore pregnancy outcomes after a miscarriage; however, these studies investigated limited outcomes such as recurrent miscarriage and PTB [15–18]. Therefore, there is a dearth of research exploring the subsequent risks of other pregnancy outcomes including preeclampsia and GDM following miscarriage or induced abortion. Our objective was therefore to investigate the risk of a broad range of adverse pregnancy outcomes such as PTB, spontaneous PTB, small for gestational age (SGA), large for gestational age (LGA), preeclampsia, and GDM according to IPI for pregnancies that follow miscarriages or induced abortions separately, with the intention of informing intrapartum care following early pregnancy loss.

## Methods

### Study design and data sources

We conducted a retrospective cohort study using 3 Norwegian national health registries: the Medical Birth Registry of Norway (birth registry) [19], the Norwegian Patient Registry (patient registry), and the General Practitioner database [20]. Using these 3 registries, we identified all registered pregnancies in Norway with an estimated date of conception between January 1, 2008 and December 31, 2016. The birth registry includes mandatory notifications on pregnancies in Norway ending after 12 gestational weeks and provided information on live births, stillbirths, miscarriages, and late induced abortions.

The patient and general practitioners’ registries provided information on induced abortions and miscarriages irrespective of gestational weeks. A detailed description of the processes for identification of miscarriage and induced abortions and data linkage procedures have been described previously [21]. In summary, we used primary and secondary diagnostic codes indicating the presence of a miscarriage or induced abortions. From the patient registry, information on miscarriages before 12 completed gestational weeks were captured using the following International Classification of Diseases version 10 (ICD-10) codes: missed abortion (O02.1); other specified abnormal products of conception (O02.8); abnormal product of conception, unspecified (O02.9); spontaneous abortion (O03); and threatened abortion (O20.0). Similarly, induced abortions were identified using the following ICD-10 codes: medical abortion (O04), other abortion (O05), and unspecified abortion (O06). In the general practitioner database, we captured information on miscarriages before 12 completed gestational weeks using the following ICPC-2 codes: bleeding in pregnancy (W03) and spontaneous abortion (W82). We only counted registrations of threatened abortion and bleeding in pregnancy codes as miscarriage or induced abortions if they did not subsequently end in a registration in the birth registry.

In this study, a fetal death at 20 gestational weeks or later or with a birthweight of 400 grams or more was considered a stillbirth, while fetal deaths prior to 20 gestational weeks with a birthweight less than 400 grams were defined as miscarriages. This study is reported as per the Strengthening the Reporting of Observational Studies in Epidemiology (STROBE) guideline [22] (S1 STROBE Checklist).

### Participants and exclusions

**Fig 1:**
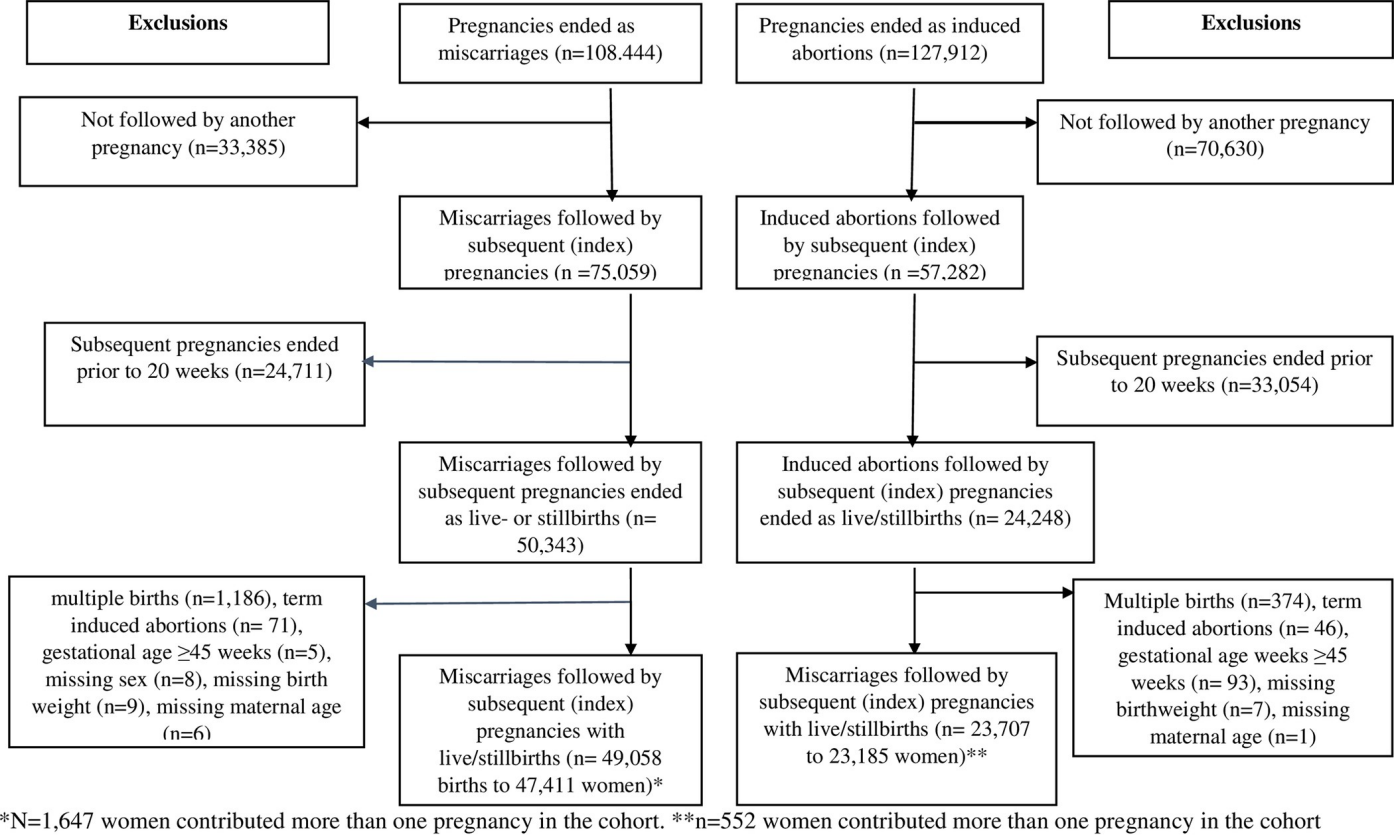
Flow chart for selection of births following miscarriages and induced abortions between 2008 and 2016 in Norway.

In the study period, there were a total of 108,444 miscarriages of which 75,059 were followed by subsequent pregnancies, and 127,912 induced abortions of which 57,282 were followed by subsequent pregnancies. After excluding pregnancies ended prior to 20 weeks, we were left with 50,343 births after miscarriages and 24,248 births after induced abortions. By further excluding multiple births, births with gestational age ≥45 weeks, late induced abortions, births with missing maternal age, and births with missing information on birthweight or offspring sex, we had a sample size of 49,058 index births after previous miscarriage and 23,707 index births after previous induced abortions. The index birth was defined as the birth after the IPI. A total of 1,647 (3.4%) women after miscarriages and 552 (2.3%) women after induced abortions contributed more than one birth in the study population (Fig 1).

### Exposure

IPI was defined as the time between the date of previous miscarriage or induced abortion and date of the conception (date of birth minus gestational age) of the subsequent live or stillbirth recorded in the birth registry. The gestational age estimation was largely based on ultrasound estimates (99%). We categorised IPI into 6 categories: <3, 3 to 5, 6 to 11, 12 to 17, 18 to 23, and ≥24 months, with 6 to 11 months as the reference category, consistent with the WHO guidelines and literature [9,10,23].

### Outcomes

We studied 6 adverse pregnancy outcomes: PTB, spontaneous PTB, SGA, LGA, preeclampsia, and GDM. [10,24–26] PTB was defined as birth occurring before 37 completed weeks of gestation. Spontaneous PTB was defined as PTB with spontaneous onset of labour. SGA and LGA were defined as a birthweight in the lowest or highest 10th percentiles, based on the gestational week and sex-specific distributions of birthweight among all births in the birth registry. Preeclampsia was defined as any registration of preeclampsia, eclampsia, or HELLP syndrome (haemolysis, elevated liver enzymes, and low platelet count). GDM was defined based on the WHO-1999 criteria when fasting plasma glucose level ≥7.0 mmol/L or glucose tolerance test of ≥7.8 mmol/L and <11.0 mmol/l [27].

### Statistical analysis

We evaluated the relationship between IPI and the pregnancy outcomes separately for births after miscarriages and births after induced abortions. We used log-binomial regression to estimate unadjusted and adjusted relative risk (aRR) and 95% CIs [17]. Robust cluster variance estimation was used to account for women who had more than one pregnancy during the study period. We adjusted for maternal age using restricted cubic splines with 5 knots (placed at the 5th, 27.5th, 50th, 72.5th, and 95th percentiles in the study population; [28]), gravidity (categorical: 1, 2, 3, or more), and year of birth (continuous) at the time of index birth. We tested for the multiplicative interaction between IPI and maternal age, and the interaction between IPI and parity, using likelihood ratio test comparing models with and without interaction terms. We calculated *e-*values for the aRR as a measure of the minimum strength of association a confounder would have to have with the exposure and outcome to explain away the observed associations. Small *e*-values indicates that little unmeasured confounding is required to explain or nullify observed effects. The converse is true of high *e*-values. The *e*-value for the lower limit of the 95% CI represents the level of confounding required to render the interval estimate null [29].

We conducted sensitivity analyses additionally adjusting for prepregnancy body mass index (BMI) (using restricted cubic splines placed at 5 knots placed at the 5th, 27.5th, 50th, 72.5th, and 95th percentiles in the study population) and smoking during pregnancy (categorical: yes/no) for the index pregnancies with information available (*n* = 27,747 for births after a miscarriage; *n* = 13,932 for births after an induced abortion). We also explored adjustment for characteristics of the miscarriage or induced abortion prior to the index pregnancy (start of the IPI), such as maternal age, gravidity, and year of end of pregnancy [30]. Since IPI categorisation that consider <6 months in the short IPI category is widely considered in the literature and for our results to be compared with the WHO recommendation, we conducted additional analysis combining the first 2 IPI categories (<3 months and 3 to 5 months). We also conducted a sensitivity analysis restricting births from women with only 1 miscarriage (*n* = 47,411) or induced abortion (*n* = 23,185) in the cohort. All analyses were conducted using STATA version 16 (Statacorp, Texas).

### Ethics statement

Ethics approval was obtained the Regional Committee for Medical and Health Research Ethics of South/East Norway (2014/404).

## Results

### Maternal characteristics at the time of birth after miscarriages and induced abortions

We included 49,058 index births after miscarriage and 23,707 index births after an induced abortion. The median maternal age at the index birth was 29 years (interquartile range (IQR) = 25 to 33 years) among women with births after a miscarriage and 28 years (IQR = 24 to 48) among women with births after an induced abortion (Tables 1 and 2).

**Table 1:** Characteristics for women with births after miscarriage and induced abortion at the time of birth after the IPI for births between 2008 and 2016 in Norway (*n* = 49,058).

Variable	Number (%)
**Maternal age (in years)**	
14–19	377 (0.8)
20–24	5,148 (10.5)
25–29	13,157 (26.8)
30–34	16,174 (33.0)
35–39	10,867 (22.1)
≥40	3,335 (6.8)
Median (IQR)	29 (25–33)
**Gravidity**	
1	19,602 (40.1)
2	14,305 (29.2)
3+	15,151 (30.8)
**Prepregnancy BMI (kg/m** ^**2**^ **)**	
<18.5	986 (2.0)
18.5–25	17,611 (35.9)
25–30	7,213 (14.7)
≥30	4,123 (8.4)
Missing	19,127 (39.0)
**Smoking during pregnancy**	
No	39,302 (80.1)
Yes	3,780 (7.7)
Missing	5,976 (12.2)
**Birth year**	
2008	238 (0.5)
2009	4,358 (8.9)
2010	5,955 (12.1)
2011	6,241 (12.7)
2012	6,330 (12.9)
2013	6,364 (12.9)
2014	6,364 (13.0)
2015	6,607 (13.5)
2016	6,585 (13.4)
**IPI in months**	
<3	17,251 (35.2)
<6	30,076 (61.3)
6–11	9,975 (20.3)
12–17	3,653 (7.5)
18–23	1,973 (4.0)
≥24	3,381 (6.9)
Median (IQR)	4 (2–9)

BMI, body mass index; IPI, interpregnancy interval; IQR, interquartile range.

**Table 2:** Characteristics for women with births after induced abortion at the time of birth after the IPI for births between 2008 and 2016 in Norway (*n* = 23,707).

Variable	Number (%)
**Maternal age (in years)**	
14–19	734 (3.1)
20–24	5,531 (23.3)
25–29	8,041 (33.9)
30–34	5,944 (25.1)
35–39	2,782 (11.7)
≥40	675 (2.9)
Median (IQR)	28 (24–48)
**Gravidity**	
1	15,082 (63.6)
2	5,233 (22.0)
3+	3,402 (14.4)
**Prepregnancy BMI (kg/m** ^**2**^ **)**	
18.5	765 (3.2)
18.5–25	9,523 (40.2)
25–30	3.277 (13.8)
≥30	1,583 (6.9)
Missing	8,559 (36.1)
**Smoking during pregnancy**	
No	16,702 (70.5)
Yes	4,017 (16.9)
Missing	2,988 (12.6)
**Birth year**	
2008	20 (0.1)
2009	933 (3.9)
2010	1,930 (8.1)
2011	2,576 (10.9)
2012	3,154 (13.3)
2013	3,455 (14.6)
2014	3,761 (15.9)
2015	3,879 (16.4)
2016	3,999 (16.9)
**IPI in months**	
<3	1,633 (6.9)
<6	4,574 (19.3)
6–11	4,244 (17.9)
12–17	3,255 (13.7)
18–23	2,540 (10.7)
≥24	9,094 (38.4)
Median (IQR)	17 (7–34)

BMI, body mass index; IPI, interpregnancy interval; IQR, interquartile range.

### Previous miscarriage

**Fig 2:**
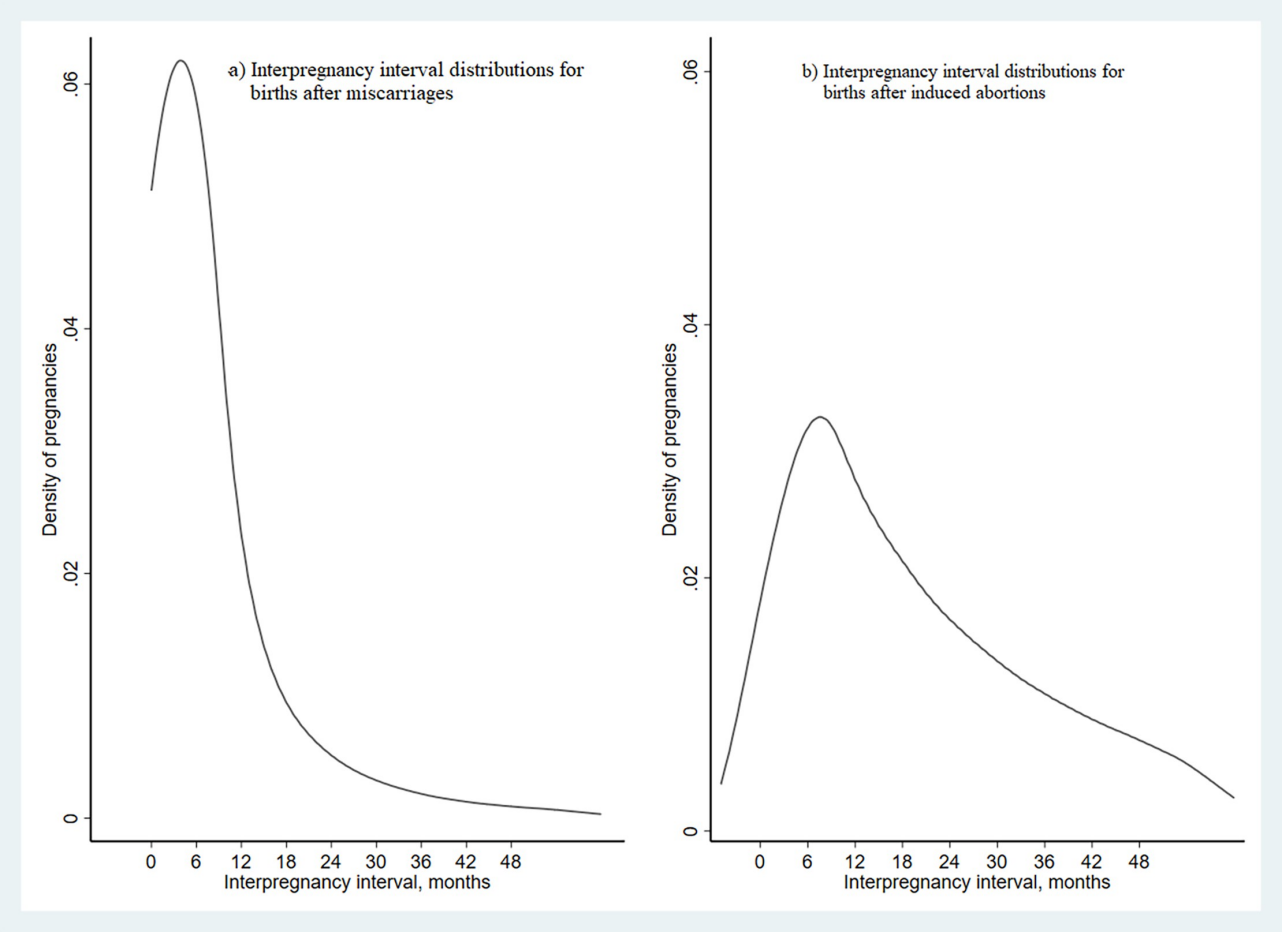
Kernel density distributions of interpregnancy interval after miscarriages and induced abortions for births between 2008 and 2016 in Norway.

The median IPI after a miscarriage was 4 months (IQR = 2 to 9 months); 61% (*n* = 30,076) conceived again within 6 months and 20% (*n* = 9,975) conceived between 6 and 11 months (Table 1 and Fig 2). In births following a previous miscarriage, there were 6.0% PTB, 3.5% spontaneous PTB, 9.5% SGA births, 10.3% LGA births, 3.2% preeclampsia, and 4.2% GDM (S1 Table).

In the adjusted analysis, women with an IPI <3 months (8.6%) (aRR 0.85, 95% CI: 0.79, 0.92, *p* < 0.01) and 3 to 5 months (9.0%) (aRR 0.89, 95% CI: 0.82, 0.96, *p* = 0.01) had a lower risk of SGA compared to women with an IPI of 6 to 11 months (10.1%). We also observed a lower risk of GDM (aRR 0.85, 95% CI: 0.75, 0.95, *p* = 0.01) among women with an IPI of <3 months (3.3%) compared to women with an IPI of 6 to 11 months (4.5%).

There was no evidence of increased risks of adverse pregnancy outcomes associated with an IPI >12 months (*p* > 0.05) except for GDM. For GDM, compared to IPI of 6 to 11 months (4.5%), there were increased risks for births after 12 to 17 months (5.8%) (aRR 1.20, 95% CI: 1.02, 1.40, *p* = 0.02), 18 to 23 months (aRR 1.24, 95% CI: 1.02, 1.50), but not for IPI ≥24 months (aRR 1.14, 95% CI: 0.97, 1.34, *p* = 0.10) (Table 3). The corresponding *e*-values were low, ranging between 1.11 and 1.83, for the associations between IPI and all adverse outcomes after previous miscarriage.

**Table 3:** IPI after previous miscarriage and risk of adverse pregnancy outcomes for births between 2008 and 2016 in Norway (*n* = 49,058).

Outcome	IPI	Number of cases (%)	RR (95% CI)	aRR (95% CI)*	*p*-value for aRR	E-value aRR** (lower 95% CI)
**PTB (*n* = 49,058)**	<3 m	1,002 (5.8)	0.94 (0.86, 1.04)	0.96 (0.87, 1.06)	0.40	1.25 (1)
	3–5 m	719 (5.6)	0.91 (0.82, 1.01)	0.92 (0.83, 1.02)	0.12	1.39 (1)
	6–11 m	615 (6.2)	Ref	Ref		Ref
	12–17 m	257 (7.0)	1.14 (0.99, 1.31)	1.14 (0.99, 1.31)	0.08	1.54 (1)
	18–23 m	150 (7.6)	1.23 (1.04, 1.45)	1.23 (1.04, 1.46)	0.02	1.76 (1.24)
	≥24 m	230 (6.8)	1.10 (0.95, 1.28)	1.14 (0.98, 1.32)	0.09	1.54 (1)
**Spontaneous PTB (*n* = 47,780)**	<3 m	592 (3.5)	0.98 (0.86, 1.11)	0.97 (0.85, 1.11)	0.64	1.21 (1)
	3–5 m	403 (3.2)	0.89 (0.78, 1.03)	0.89 (0.78, 1.03)	0.11	1.50 (1)
	6–11 m	350 (3.6)	Ref	Ref		Ref
	12–17 m	137 (3.9)	1.08 (0.89, 1.31)	1.08 (0.89, 1.31)	0.43	1.37 (1)
	18–23 m	86 (4.5)	1.25 (0.99, 1.57)	1.26 (1.00, 1.59)	0.05	1.83 (1)
	≥24 m	127 (3.9)	1.07 (0.88, 1.31)	1.12 (0.92, 1.37)	0.26	1.49 (1)
**SGA (*n* = 49,058)**	<3 m	1,483 (8.6)	0.85 (0.79, 0.92)	0.85 (0.79, 0.92)	0.00	1.63 (1.39)
	3–5 m	1,157 (9.0)	0.89 (0.82, 0.97)	0.89 (0.82, 0.96)	0.01	1.50 (1.25)
	6–11 m	1,008 (10.1)	Ref	Ref		Ref
	12–17 m	420 (11.5)	1.14 (1.02, 1.27)	1.13 (1.02, 1.26)	0.03	1.51 (1.16)
	18–23 m	197 (10.0)	0.99 (0.85, 1.14)	0.97 (0.84, 1.12)	0.70	1.21 (1)
	≥24 m	389 (11.5)	1.14 (1.02, 1.27)	1.09 (0.97, 1.21)	0.15	1.40 (1)
**LGA (*n* = 49,058)**	<3 m	1,809 (10.5)	1.02 (0.95, 1.10)	1.04 (0.97, 1.12)	0.30	1.24 (1)
	3–5 m	1,312 (10.2)	1.00 (0.93, 1.08)	1.01 (0.93, 1.09)	0.82	1.11 (1)
	6–11 m	1,021 (10.2)	Ref	Ref		Ref
	12–17 m	390 (10.7)	1.04 (0.93, 1.16)	1.05 (0.94, 1.17)	0.34	1.28 (1)
	18–23 m	197 (10.0)	0.98 (0.84, 1.13)	0.99 (0.86, 1.15)	0.92	1.11 (1)
	≥24 m	314 (9.3)	0.91 (0.80, 1.03)	0.95 (0.85, 1.08)	0.45	1.29 (1)
**Preeclampsia (*n* = 49,058)**	<3 m	512 (3.0)	0.91 (0.80, 1.05)	0.93 (0.81, 1.07)	0.29	1.36 (1)
	3–5 m	391 (3.1)	0.94 (0.81, 1.08)	0.95 (0.82, 1.09)	0.46	1.29 (1)
	6–11 m	324 (3.4)	Ref	Ref		Ref
	12–17 m	133 (3.7)	1.12 (0.92, 1.37)	1.11 (0.91, 1.35)	0.32	1.46 (1)
	18–23 m	72 (3.6)	1.12 (0.87, 1.44)	1.11 (0.86, 1.42)	0.43	1.46 (1)
	≥24 m	114 (3.4)	1.04 (0.84, 1.28)	1.01 (0.82, 1.25)	0.92	1.11 (1)
**GDM (*n* = 49,058)**	<3 m	571 (3.3)	0.74 (0.66, 0.84)	0.85 (0.75, 0.95)	0.01	1.67 (1.29)
	3–5 m	508 (4.0)	0.89 (0.79, 1.00)	0.95 (0.84, 1.08)	0.43	1.29 (1)
	6–11 m	446 (4.5)	Ref	Ref		Ref
	12–17 m	211 (5.8)	1.29 (1.10, 1.51)	1.20 (1.02, 1.40)	0.02	1.69 (1.16)
	18–23 m	122 (6.2)	1.38 (1.14, 1.68)	1.24 (1.02, 1.50)	0.03	1.79 (1.16)
	≥24 m	216 (6.4)	1.43 (1.22, 1.67)	1.14 (0.97, 1.34)	0.10	1.54 (1)

aRR, adjusted relative risk; CI, confidence interval; GDM, gestational diabetes mellitus; IPI, interpregnancy interval; LGA, large for gestational age; PTB, preterm birth; RR, relative risk; SGA, small for gestational age.

*Adjusted for maternal age, gravidity, and year of birth at the time of birth after interval. For maternal age, we used restricted cubic splines with 5 knots placed at the 5th, 27.5th, 50th, 72.5th, and 95th percentiles in the study population, which corresponds to 21, 26, 30, 33, and 40 years.

**E-values for unmeasured confounding for the association between IPI after miscarriage and induced abortion and adverse pregnancy outcomes.

### Previous induced abortion

The median IPI after a previous induced abortion was 17 months (IQR = 7 to 34 months), where 19% (*n* = 4,574) of births were conceived within 6 months, and 18% (*n* = 4,244) were conceived between 6 and 11 months (Table 2 and Fig 2). There were 5.5% PTB, 3.2% spontaneous PTB, 10.8% SGA births, 8.8% LGA births, 2.8% preeclampsia, and 3.2% GDM among births among births following induced abortions (S2 Table).

Compared with births following 6 to 11 months of IPI, there was a modest increased risk of SGA after an IPI of <3 months with a confidence interval spanning the null value (aRR 1.16, 95% CI: 0.99, 1.37, *p* = 0.07), and a modest decreased risk of LGA among those with an IPI of 3 to 5 months (aRR 0.84, 95% CI: 0.72, 0.98, *p* = 0.03). For IPI >12 months, there was no evidence of risk of adverse pregnancy outcomes (Table 4). Corresponding *e*-values were low, ranging between 1.21 and ≤2.55, for the associations between IPI and adverse outcomes after previous induced abortion.

**Table 4:** IPI after previous induced abortion and risk of adverse pregnancy outcomes for births between 2008 and 2016 in Norway (*n* = 23,707).

Outcome	IPI	Number of cases (%)	RR (95% CI)	aRR (95% CI)*	*p*-value for aRR	E-value aRR** (lower 95% CI)
**PTB (*n* = 23,707)**	<3 m	110 (6.7)	1.20 (0.97, 1.49)	1.17 (0.94, 1.46)	0.15	1.62 (1)
	3–5 m	157 (5.3)	0.95 (0.78, 1.16)	0.94 (0.77, 1.14)	0.53	1.32 (1)
	6–11 m	238 (5.6)	Ref	Ref		Ref
	12–17 m	150 (4.6)	0.82 (0.67, 1.00)	0.84 (0.69, 1.02)	0.08	1.67 (1)
	18–23 m	134 (5.3)	0.94 (0.77, 1.16)	0.97 (0.79, 1.20)	0.80	1.21 (1)
	≥24 m	506 (5.6)	1.00 (0.85, 1.15)	1.10 (0.94, 1.30)	0.22	1.43 (1)
**Spontaneous PTB (*n* = 23,163)**	<3 m	62 (3.9)	1.15 (0.86, 1.54)	1.13 (0.85, 1.52)	0.41	1.51 (1)
	3–5 m	102 (3.5)	1.04 (0.81, 1.34)	1.03 (0.80, 1.32)	0.83	1.21 (1)
	6–11 m	141 (3.4)	Ref	Ref		Ref
	12–17 m	67 (2.1)	0.62 (0.47, 0.83)	0.63 (0.47, 0.84)	0.00	2.55 (1.56)
	18–23 m	84 (3.4)	0.99 (0.76, 1.29)	1.02 (0.78, 1.33)	0.90	1.16 (1)
	≥24 m	295 (3.3)	0.98 (0.80, 1.19)	1.06 (0.86, 1.31)	0.56	1.31 (1)
**SGA (*n* = 23,707)**	<3 m	188 (11.5)	1.16 (0.98, 1.36)	1.16 (0.99, 1.37)	0.07	1.59 (1)
	3–5 m	318 (10.8)	1.08 (0.95, 1.24)	1.09 (0.95, 1.25)	0.23	1.40 (1)
	6–11 m	423 (10.0)	Ref	Ref		Ref
	12–17 m	363 (11.2)	1.12 (0.98, 1.28)	1.12 (0.98, 1.28)	0.09	1.49 (1)
	18–23 m	269 (10.6)	1.06 (0.92, 1.23)	1.07 (0.93, 1.24)	0.37	1.34 (1)
	≥24 m	997 (11.0)	1.10 (0.99, 1.23)	1.12 (1.00, 1.26)	0.05	1.49 (1)
**LGA (*n* = 23,707)**	<3 m	152 (9.3)	0.99 (0.83, 1.18)	0.98 (0.82, 1.17)	0.80	1.16 (1)
	3–5 m	234 (8.0)	0.84 (0.72, 0.99)	0.84 (0.72, 0.98)	0.03	1.67 (1.16)
	6–11 m	400 (9.4)	Ref	Ref		Ref
	12–17 m	307 (9.4)	1.00 (0.87, 1.15)	1.00 (0.87, 1.15)	0.99	1.00 (1)
	18–23 m	205 (8.1)	0.86 (0.73, 1.01)	0.85 (0.73, 1.01)	0.06	1.63 (1)
	≥24 m	811 (8.9)	0.95 (0.84, 1.06)	0.95 (0.84, 1.07)	0.41	1.29 (1)
**Preeclampsia (*n* = 23,707)**	<3 m	50 (3.1)	1.13 (0.81, 1.57)	1.15 (0.83, 1.60)	0.40	1.57 (1)
	3–5 m	80 (2.7)	1.00 (0.76, 1.33)	1.02 (0.77, 1.35)	0.90	1.16 (1)
	6–11 m	115 (2.7)	Ref	Ref		Ref
	12–17 m	80 (2.5)	0.91 (0.68, 1.20)	0.90 (0.68, 1.20)	0.49	1.46 (1)
	18–23 m	63 (2.5)	0.92 (0.69, 1.24)	0.91 (0.67, 1.23)	0.53	1.43 (1)
	≥24 m	277 (3.1)	1.12 (0.91, 1.39)	1.09 (0.87, 1.36)	0.46	1.40 (1)
**GDM (*n* = 23,707)**	<3 m	36 (2.2)	0.75 (0.52, 1.08)	0.75 (0.52, 1.08)	0.13	2.00 (1)
	3–5 m	76 (2.6)	0.88 (0.66, 1.16)	0.88 (0.67, 1.16)	0.37	1.53 (1)
	6–11 m	125 (3.0)	Ref	Ref		Ref
	12–17 m	98 (3.0)	1.02 (0.79, 1.33)	0.97 (0.75, 1.25)	0.81	1.21 (1)
	18–23 m	63 (2.5)	0.84 (0.62, 1.14)	0.79 (0.59, 1.06)	0.12	1.85 (1)
	≥24 m	354 (3.9)	1.32 (1.08, 1.62)	1.04 (0.85, 1.28)	0.72	1.24 (1)

aRR, adjusted relative risk; CI, confidence interval; IPI, interpregnancy interval; GDM, gestational diabetes mellitus; LGA, large for gestational age; PTB, preterm birth; RR, relative risk; SGA, small for gestational age.

*Adjusted for maternal age, gravidity, and year of birth at the time of birth after interval. For maternal age, we used restricted cubic splines with 5 knots placed at the 5th, 27.5th, 50th, 72.5th, and 95th percentiles in the study population, which corresponds to 20, 25, 28, 32, and 38 years.

**E-values for unmeasured confounding for the association between IPI after miscarriage and induced abortion and adverse pregnancy outcomes.

### Sensitivity analysis

Additional adjustment for smoking during pregnancy and prepregnancy BMI did not alter our conclusions (S3 and S4 Tables). Our results adjusting for covariates measured at the time of miscarriage or induced abortion (before interval) provided consistent findings (S5 and S6 Tables). Our analysis combining <3 months and 3 to 5 months of IPI into a <6 months category showed consistent results with estimates for IPI <3 months category (S7 and S8 Tables). Our results restring births from women with only 1 miscarriage or induced abortion in the cohort did not change our results (S9 and S10 Tables)

## Discussion

We used a Norwegian national registry-linkage to investigate the association between IPI following a miscarriage or induced abortion with adverse pregnancy outcomes in the subsequent birth. Contrary to the current WHO recommendations advising women to wait a minimum of 6 months after miscarriages or induced abortions [9], we found no evidence of elevated risk of PTB, spontaneous PTB, LGA, or preeclampsia among women with a very short (<3) or short (<6 months) IPIs after a miscarriages or induced abortions. Rather, we found that the risk of SGA was lower among women with births following an IPI of <3 months and 3 to 5 months after miscarriage. We also found a lower risk of GDM among women with very short (<3 months) IPI after miscarriage. There was no evidence of higher risks of adverse pregnancy outcomes for IPIs >12 months after miscarriages or induced abortions except for a higher risk of GDM for births after miscarriages with IPIs between 12 and 23 months.

Our study is consistent with previous studies from Scotland [10] and the United States [15–17], which reported no difference or reduced risk of adverse pregnancy outcomes following short (<6 months) IPI after a previous miscarriage. Although not directly comparable to our study due to only including births after first trimester miscarriages and having a very small sample size (*n* = 107), our results were consistent with a study from Israel that indicated that conception shortly after a spontaneous miscarriage was not associated with adverse maternal or neonatal outcomes [31]. Our results differ from a large cohort study conducted in Latin America, which did not distinguish between miscarriages and induced abortions reporting an elevated risk of adverse pregnancy outcomes among women with an IPI of <6 months [8]. On the other hand, another study from the US considering births after miscarriages and induced abortions together showed a reduced risk of PTB [11]. Previous studies from Finland [12] and China [32] investigating the effect of IPI after induced abortions showed increased risks of PTB and SGA after short (<6 months) IPI. Unlike these studies, we observed no evidence of elevated risks of either PTB or SGA according to IPI following an induced abortion. Apart from one study from Finland [12], our study was not in agreement with previous studies that reported elevated risks of adverse pregnancy outcomes for those pregnancies with long (>24 months) IPI after miscarriages as compared to IPI 6 to 11 months [10,33,34]. As our study included women with at least 2 pregnancies occurring in less than a decade, we might not have observed adequate number of births with longer IPIs to observe any associations. There were also some differences between our study and previous studies where some studies used 18 to 23 months as reference [8,11,32], included smaller sample sizes [12,15,17,18,32], and relied on self-reported data [8,32].

Although the proportion of short (<6 months) IPI were generally high following both miscarriages and induced abortions, the proportion of short IPI that followed miscarriages (61%) were much higher than the proportion of short IPI that followed induced abortions (19%). A previous study reported over one-third of births (37%) following a stillbirth were conceived within the first 6 months [35]. However, for IPIs after live births, a study that involved 4 high-income countries including Norway showed that less than 5% of conceptions following live births occurred in the first 6 months [36]. Our findings, in combination with the previous findings of Regan and colleagues [35] indicate that women with early and late pregnancy losses (miscarriages or stillbirths) attempt pregnancy more quickly than those women whose pregnancies ended with live births. On the other hand, IPIs after induced abortions could be relatively longer if previous terminations resulted from unintended pregnancy, and, hence, these women could adopt contraceptive methods as part of routine postabortal care [37].

Our findings suggested that IPI <6 months following miscarriages may not increase the risks of adverse outcomes in the next pregnancy. One possible explanation would be that pregnancies conceived shortly after a miscarriage are more likely to be intended, and, hence, these women may seek health services with the aim of avoiding the previous unfavourable experience [10]. The reduced risk of SGA after very short (<3 months) and short (3 to 5 months) IPI in our study may support the previous hypothesis suggesting women who were able to conceive quickly after miscarriages might have better fecundity reflecting women’s high reproductive fitness in comparison to women with longer IPI [16]. Despite the assumption that women with previous pregnancies resulting in a live birth could have had nutritional depletions, which are mostly occurring in the second trimester and early postpartum period through breastfeeding [38], it is plausible that women with miscarriages would not reach at a point when nutritional depletion starts as most miscarriages usually occur in the first 12 weeks [39,40].

A large study conducted in Latin America indicated an elevated risk of adverse pregnancy outcomes despite not separating miscarriage from induced abortion [8]. The authors hypothesized that their results could be attributed to reproductive tract infections due to the possibility of unsafe abortions, which may lead to negative consequences to the growing fetus in the subsequent pregnancy. However, our results indicated no elevated risk of adverse pregnancy outcomes in those pregnancies that followed induced abortions. This could be due to the provision of safe abortion services free of cost in the public health system in Norway, and, hence, the risks of infections are minimal [37]. Studies also indicated that uterine infections are mostly associated with surgical abortions than medical terminations. In Norway, medical abortions comprising more than 80% of all abortions conducted in 2013, and 95% of these terminations were performed in the first trimester [41]. Since 2009, medical abortion services were further expanded for women to get access to self-administered misoprostol to use at home for pregnancies terminations up to 12 weeks of gestation [42].

Previous studies did not assess the association between previous miscarriage and risk of GDM in the subsequent pregnancy according to interpregnancy interval. Two Chinese studies [43,44] reported that women who had previous miscarriage had a higher risk for developing gestational diabetes during subsequent pregnancies, although their studies did not evaluate IPI specifically. In our study, while we observed an increased risk of risk of GDM for births between 12 and 23 months of IPI, we found a reduced risk of GDM for births after a very short (<3 months) IPI. The underlying mechanisms for the associations are unclear and warrant further investigations. Yang and colleagues [44] speculated that the association between previous miscarriages and GDM might have occurred due to common risk factors or shared pathological mechanisms.

The strengths of our study are its ability to provide comprehensive information of early miscarriages and induced abortions from the combination of national health registries in Norway, in addition to be able to distinguish the risks of adverse pregnancy outcomes according to IPI after a miscarriage and an induced abortion, which was not possible in most settings [8,11]. Unlike most previous studies [10,26,34] that investigated IPIs of <6 months after miscarriage or induced abortions, but not all [15,16], our study investigated the effect of IPI for very short (<3 months) and short (3 to 5 months) IPIs. To allow comparison with literature and WHO recommendation, we also estimated risk of adverse pregnancy outcomes for IPI <6 months, although the results broadly concurred with the estimates for IPI <3 months. Although the gestational age at previous miscarriage or induced abortion affects the IPI, we did not have information on exact gestational age for those miscarriages or induced abortions occurred before 12 weeks, which comprised 99% of miscarriages and 96% of induced abortions. However, we did have information on gestational age at miscarriages or induced abortions after 12 weeks, and, hence, the risk of misclassification of exposure is minimal for these pregnancies. With regard to the timing of confounder measurement, it has recently been recommended to adjust confounders for measures prior to IPI (i.e., at the time of the miscarriage or induced abortion), as these are the characteristics that may be associated both with IPI and adverse pregnancy outcomes [30,45]. Our sensitivity analysis adjusting for maternal age, gravidity, and year at the time of the miscarriage or induced abortion indicated no significant difference in the risk estimates from the main results. However, since smoking and prepregnancy BMI information were only available for pregnancies registered in the birth registry, we were not able to adjust for these variables at the time of miscarriage or induced abortion. These results were not unexpected because most of the pregnancies occurred within 1 year of miscarriage or induced abortion, and, hence, maternal characteristics including maternal age and socioeconomic status are unlikely to change significantly. Moreover, inherent to retrospective registry-based studies, we did not have information on potential confounders variables. For example, while pregnancy intention and health-seeking behaviour are likely to vary between women with longer pregnancies [46–48] but were unfortunately not available. Furthermore, we only had information on miscarriages that resulted in contact with the healthcare system. Since our study employed data from a single high-income country with better healthcare services, our results could not be generalisable to other settings with different population.

In conclusion, our study found that that conceiving within 6 months after a miscarriage or an induced abortion was not associated with increased risks of adverse pregnancy outcomes. In combination with previous research, these results suggest that women could attempt pregnancy soon after a previous miscarriage or induced abortion without increasing perinatal health risks. Our results do not support current international recommendations to wait at least 6 months after miscarriages or induced abortions.

## Supporting information

S1 STROBE Checklist STROBE checklist. (DOCX)

S1 Table Adverse pregnancy outcomes after miscarriages between 2008 and 2016 in Norway (*n* = 49,058). GDM, gestational diabetes mellitus; LGA, large for gestational age; PTB, preterm birth; SGA, small for gestational age. *Births with nonspontaneous preterm outcomes were excluded when defining spontaneous PTB.
(DOCX)

S2 Table Adverse pregnancy outcomes after induced abortion between 2008 and 2016 in Norway (*n* = 23,707). GDM, gestational diabetes mellitus; LGA, large for gestational age; PTB, preterm birth; SGA, small for gestational age. *Births with nonspontaneous preterm outcomes were excluded when defining spontaneous PTB.
(DOCX)

S3 Table Sensitivity analysis—IPI after previous miscarriage and risk of adverse pregnancy outcomes with complete information on maternal smoking during pregnancy and prepregnancy BMI for births between 2008 and 2016 in Norway (*n* = 27,747). aRR, adjusted relative risk; BMI, body mass index; CI, confidence interval; GDM, gestational diabetes mellitus; IPI, interpregnancy interval; LGA, large for gestational age; PTB, preterm birth; RR, relative risk; SGA, small for gestational age. *Births with nonspontaneous preterm outcomes were excluded when defining spontaneous PTB. **Adjusted for maternal age, gravidity, year of birth, maternal smoking during pregnancy, and prepregnancy BMI at the time of birth after interval. For maternal age and prepregnancy BMI variables, we used restricted cubic splines with 5 knots placed at the 5th, 27.5th, 50th, 72.5th, and 95th percentiles in the study population, which corresponds to 21, 26, 30, 33, and 40 years for maternal age, and 19, 21, 24, 27, and 34 kg/m^2^ for prepregnancy BMI.
(DOCX)

S4 Table Sensitivity analysis—IPI after previous induced abortion and risk of adverse pregnancy outcomes with complete information on maternal smoking during pregnancy and prepregnancy BMI for births between 2008 and 2016 in Norway (*n* = 13,932). aRR, adjusted relative risk; BMI, body mass index; CI, confidence interval; GDM, gestational diabetes mellitus; IPI, interpregnancy interval; LGA, large for gestational age; PTB, preterm birth; RR, relative risk; SGA, small for gestational age. *Births with nonspontaneous preterm outcomes were excluded when defining spontaneous PTB. **Adjusted for maternal age, parity, year of birth, maternal smoking during pregnancy, and prepregnancy BMI at the time of birth after interval. For maternal age and prepregnancy BMI variables, we used restricted cubic splines with 5 knots placed at the 5th, 27.5th, 50th, 72.5th, and 95th percentiles in the study population, which corresponds to 20, 25, 28, 32, and 38 years for maternal age, and 18, 21, 23, 26, and 33 kg/m^2^ for prepregnancy BMI.
(DOCX)

S5 Table Sensitivity analysis—aRR for the association between IPI after a miscarriage and adverse pregnancy outcomes adjusted for covariates prior to IPI (*n* = 49,058). aRR, adjusted relative risk; BMI, body mass index; CI, confidence interval; GDM, gestational diabetes mellitus; IPI, interpregnancy interval; LGA, large for gestational age; PTB, preterm birth; RR, relative risk; SGA, small for gestational age. *Births with nonspontaneous preterm outcomes were excluded when defining spontaneous PTB. **Adjusted for maternal age, gravidity, and year of birth at the time of miscarriage (before interval). For maternal age, we used restricted cubic splines with 5 knots placed at the 5th, 27.5th, 50th, 72.5th, and 95th percentiles in the study population, which corresponds to 21, 26, 30, 33, and 40 years for births after a miscarriage.
(DOCX)

S6 Table Sensitivity analysis—aRR for the association between IPI after an induced abortion and adverse pregnancy outcomes adjusted for covariates prior to IPI (*n* = 23,707). aRR, adjusted relative risk. BMI, body mass index; CI, confidence interval; GDM, gestational diabetes mellitus; IPI, interpregnancy interval; LGA, large for gestational age; PTB, preterm birth; RR, relative risk; SGA, small for gestational age. *Births with nonspontaneous preterm outcomes were excluded when defining spontaneous PTB. **Adjusted for maternal age, gravidity, and year of birth at the time of miscarriage (before interval). For maternal age, we used restricted cubic splines with 5 knots placed at the 5th, 27.5th, 50th, 72.5th, and 95th percentiles in the study population, which corresponds to 18, 22, 25, 29, and 36 for births after an induced abortion.
(DOCX)

S7 Table Sensitivity analysis—IPI after previous miscarriage and risk of adverse pregnancy outcomes accounting <6 months of IPI category (*n* = 49,058). aRR, adjusted relative risk. BMI, body mass index; CI, confidence interval; GDM, gestational diabetes mellitus; IPI, interpregnancy interval; LGA, large for gestational age; PTB, preterm birth; RR, relative risk; SGA, small for gestational age. *Births with nonspontaneous preterm outcomes were excluded when defining spontaneous PTB. **Adjusted for maternal age, gravidity, and year of birth at the time of birth after interval. For maternal age, we used restricted cubic splines with 5 knots placed at the 5th, 27.5th, 50th, 72.5th, and 95th percentiles in the study population, which corresponds to 21, 26, 30, 33, and 40 years.
(DOCX)

S8 Table Sensitivity analysis—IPI after previous induced abortion and risk of adverse pregnancy outcomes accounting <6 months of IPI category (*n* = 23,707). aRR, adjusted relative risk; BMI, body mass index; CI, confidence interval; GDM, gestational diabetes mellitus; IPI, interpregnancy interval; LGA, large for gestational age; PTB, preterm birth; RR, relative risk; SGA, small for gestational age. *Births with nonspontaneous preterm outcomes were excluded when defining spontaneous PTB. **Adjusted for maternal age, gravidity, and year of birth at the time of birth after interval. For maternal age, we used restricted cubic splines with 5 knots placed at the 5th, 27.5th, 50th, 72.5th, and 95th percentiles in the study population, which corresponds to 20, 25, 28, 32, and 38 years.
(DOCX)

S9 Table Sensitivity analysis—IPI after previous miscarriages and risk of adverse pregnancy outcomes among births from women with only 1 miscarriage in the cohort (*n* = 47,411). aRR, adjusted relative risk; BMI, body mass index; CI, confidence interval; GDM, gestational diabetes mellitus; IPI, interpregnancy interval; LGA, large for gestational age; PTB, preterm birth; RR, relative risk; SGA, small for gestational age. *Births with nonspontaneous preterm outcomes were excluded when defining spontaneous PTB. *Adjusted for maternal age, gravidity, and year of birth at the time of birth after interval. For maternal age, we used restricted cubic splines with 5 knots placed at the 5th, 27.5th, 50th, 72.5th, and 95th percentiles in the study population, which corresponds to 21, 26, 30, 33, and 40 years. **E-values for unmeasured confounding for the association between IPI after miscarriage and induced abortion and adverse pregnancy outcomes.
(DOCX)

S10 Table Sensitivity analysis—IPI after previous induced abortion and risk of adverse pregnancy outcomes among births from women with only 1 induced abortion in the cohort (*n* = 23,185). aRR, adjusted relative risk; BMI, body mass index; CI, confidence interval; GDM, gestational diabetes mellitus; IPI, interpregnancy interval; LGA, large for gestational age; PTB, preterm birth; RR, relative risk; SGA, small for gestational age. *Births with nonspontaneous preterm outcomes were excluded when defining spontaneous PTB. **Adjusted for maternal age, gravidity, and year of birth at the time of birth after interval. For maternal age, we used restricted cubic splines with 5 knots placed at the 5th, 27.5th, 50th, 72.5th, and 95th percentiles in the study population, which corresponds to 20, 25, 28, 32, and 38 years. **E-values for unmeasured confounding for the association between IPI after miscarriage and induced abortion and adverse pregnancy outcomes.
(DOCX)

## Abbreviations

aRR: adjusted relative risk
BMI: body mass index
CI: confidence interval
GDM: gestational diabetes mellitus
HELLP: haemolysis, elevated liver enzymes, and low platelet count
ICD: International Classification of Diseases
IPI: interpregnancy interval
IQR: interquartile range
LBW: low birth weight
LGA: large for gestational age
OR: odds ratio
PTB: preterm birth
SGA: small for gestational age
WHO: World Health Organization

## References

[1] Magnus MC, Wilcox AJ, Morken N-H, Weinberg CR, Håberg SE. Role of maternal age and pregnancy history in risk of miscarriage: prospective register based study. BMJ. 2019;364:l869. doi: 10.1136/bmj.l869 30894356 PMC6425455

[2] Adolfsson A, Larsson P-G. Cumulative incidence of previous spontaneous abortion in Sweden in 1983–2003: a register study. Acta Obstet Gynecol Scand. 2006;85(6):741–747. doi: 10.1080/00016340600627022 16752269

[3] Andersen A-MN, Wohlfahrt J, Christens P, Olsen J, Melbye M. Maternal age and fetal loss: population based register linkage study. BMJ. 2000;320(7251):1708–1712. doi: 10.1136/bmj.320.7251.1708 10864550 PMC27416

[4] Guttmacher Institute. Unintended Pregnancy and Abortion Worldwide: Guttmacher Institute New York, NY; 2020 [2021 Apr 29]. Available from: https://www.guttmacher.org/fact-sheet/induced-abortion-worldwide.

[5] Toffol E, Koponen P, Partonen T. Miscarriage and mental health: results of two population-based studies. Psychiatry Res. 2013;205(1–2):151–158. Epub 2012/09/19. doi: 10.1016/j.psychres.2012.08.029 22985545

[6] Biggs MA, Gould H, Foster DG. Understanding why women seek abortions in the US. BMC Womens Health. 2013;13(1). doi: 10.1186/1472-6874-13-29 23829590 PMC3729671

[7] Chae S, Desai S, Crowell M, Sedgh G. Reasons why women have induced abortions: a synthesis of findings from 14 countries. Contraception. 2017;96(4):233–241Epub 2017/07/08. doi: 10.1016/j.contraception.2017.06.014 28694165 PMC5957082

[8] Conde-Agudelo A, Belizan J, Breman R, Brockman S, Rosas-Bermudez A. Effect of the interpregnancy interval after an abortion on maternal and perinatal health in Latin America. Int J Gynaecol Obstet. 2005;89:S34–S40. doi: 10.1016/j.ijgo.2004.08.003 15820366

[9] World Health Organization. Report of a WHO technical consultation on birth spacing: Geneva, Switzerland 13–15 June 2005. World Health Organization; 2007. Available from: https://apps.who.int/iris/handle/10665/69855.

[10] Love ER, Bhattacharya S, Smith NC, Bhattacharya S. Effect of interpregnancy interval on outcomes of pregnancy after miscarriage: retrospective analysis of hospital episode statistics in Scotland. BMJ. 2010;341:c3967. doi: 10.1136/bmj.c3967 20688842 PMC2917004

[11] Shachar BZ, Mayo JA, Lyell DJ, Baer RJ, Jeliffe-Pawlowski LL, Stevenson DK, et al. Interpregnancy interval after live birth or pregnancy termination and estimated risk of preterm birth: a retrospective cohort study. BJOG 2016;123(12):2009–17. doi: 10.1111/1471-0528.14165 27405702

[12] Männistö J, Bloigu A, Mentula M, Gissler M, Heikinheimo O, Niinimäki M. Interpregnancy Interval After Termination of Pregnancy and the Risks of Adverse Outcomes in Subsequent Birth. Obstet Gynecol. 2017;129(2):347–354. doi: 10.1097/AOG.0000000000001836 28079768

[13] Hanley GE, Hutcheon JA, Kinniburgh BA, Lee L. Interpregnancy Interval and Adverse Pregnancy Outcomes: An Analysis of Successive Pregnancies. Obstet Gynecol. 2017;129(3):408–415. Epub 2017/02/09. doi: 10.1097/AOG.0000000000001891 28178044

[14] Gebremedhin AT, Regan AK, Ball S, Betrán AP, Foo D, Gissler M, et al. Interpregnancy interval and hypertensive disorders of pregnancy: A population-based cohort study.Paediatr Perinat Epidemiol 2021 Jul;35(4):404–414. doi: 10.1111/ppe.12668 32189375

[15] Wong LF, Schliep KC, Silver RM, Mumford SL, Perkins NJ, Ye A, et al. The effect of a very short interpregnancy interval and pregnancy outcomes following a previous pregnancy loss. Am J Obstet Gynecol. 2015;212(3):375.e1–375.e11. doi: 10.1016/j.ajog.2014.09.020 25246378 PMC4346440

[16] Sundermann AC, Hartmann KE, Jones SH, Torstenson ES, Velez Edwards DR. Interpregnancy Interval After Pregnancy Loss and Risk of Repeat Miscarriage. Obstet Gynecol. 2017;130(6):1312–1318. doi: 10.1097/AOG.0000000000002318 29112656 PMC5709156

[17] Makhlouf MA, Clifton RG, Roberts JM, Myatt L, Hauth JC, Leveno KJ, et al. Adverse pregnancy outcomes among women with prior spontaneous or induced abortions. Am J Perinatol. 2014;31(9):765–772. doi: 10.1055/s-0033-1358771 24347257 PMC4061262

[18] Goldstein RRP, Croughan MS, Robertson PA. Neonatal outcomes in immediate versus delayed conceptions after spontaneous abortion: A retrospective case series. Am J Obstet Gynecol 2002;186(6):1230–6. 10.1067/mob.2002.123741. 12066103

[19] Irgens L. The Medical Birth Registry of Norway; a source for epidemiological and clinical research. Scand J Rheumatol. 1998;27(sup107):105–108. 10.1080/03009742.1998.11720780 9759145

[20] Bakken IJ, Ariansen AM, Knudsen GP, Johansen KI, Vollset SE. The Norwegian Patient Registry and the Norwegian Registry for Primary Health Care: Research potential of two nationwide health-care registries. Scand J Public Health. 2020;48(1):49–55. doi: 10.1177/1403494819859737 31288711

[21] Magnus MC, Havdahl A, Morken N-H, Wensaas K-A, Wilcox AJ, Håberg SE. Risk of miscarriage in women with psychiatric disorders. Br J Psychiatry 2021:1–6. doi: 10.1192/bjp.2020.259 33448259 PMC7611718

[22] Von Elm E, Altman DG, Egger M, Pocock SJ, Gøtzsche PC, Vandenbroucke JP. The Strengthening the Reporting of Observational Studies in Epidemiology (STROBE) statement: guidelines for reporting observational studies. Bull World Health Organ. 2007 Nov;85(11):867–72. doi: 10.2471/blt.07.045120 18038077 PMC2636253

[23] Kangatharan C, Labram S, Bhattacharya S. Interpregnancy interval following miscarriage and adverse pregnancy outcomes: systematic review and meta-analysis. Hum Reprod Update. 2017;23(2):221–231. doi: 10.1093/humupd/dmw043 27864302

[24] Holmes HJ, Lo JY, McIntire DD, Casey BM. Prediction of diabetes recurrence in women with class A(Diet-Treated) gestational diabetes. Am J Perinatol. 2010;27(1):47–52. doi: 10.1055/s-0029-1241733 19806532

[25] Gebremedhin AT, Regan AK, Ball S, Betrán AP, Foo D, Gissler M, et al. Interpregnancy interval and hypertensive disorders of pregnancy: A population-based cohort study. Paediatr Perinat Epidemiol. 2020. doi: 10.1111/ppe.12668 32189375

[26] Conde-Agudelo A, Belizán JM, Norton MH, Rosas-Bermúdez A. Effect of the interpregnancy interval on perinatal outcomes in Latin America. Obstet Gynecol. 2005;106(2):359–366. doi: 10.1097/01.AOG.0000171118.79529.a3 16055588

[27] World Health Organization. Definition, diagnosis and classification of diabetes mellitus and its complications: report of a WHO consultation. Part 1, Diagnosis and classification of diabetes mellitus. World Health Organization; 1999.

[28] Harrell FE. Regression modeling strategies: with applications to linear models, logistic regression, and survival analysis: Springer; 2001.

[29] VanderWeele TJ, Ding P, Mathur M. Technical Considerations in the Use of the E-Value. J Causal Inference. 2019;7(2). doi: 10.1515/jci-2018-0007

[30] Schummers L, Hutcheon JA, Norman WV, Liauw J, Bolatova T, Ahrens KA. Short interpregnancy interval and pregnancy outcomes: How important is the timing of confounding variable ascertainment? Paediatr Perinat Epidemiol. 2021 Jul;35(4):428–437. 10.1111/ppe.12716. 33270912

[31] Reicher L, Gamzu R, Fouks Y, Isakov O, Yogev Y, Maslovitz S. The effects of a postmiscarriage menstrual period prior to reconceiving. Am J Obstet Gynecol. 2020;223(3):444.e1-.e5. doi: 10.1016/j.ajog.2020.06.051 32883452

[32] Huo XX, Gao ES, Cheng YM, Luo L, Liang H, Huang GY, et al. Effect of interpregnancy interval after a mifepristone-induced abortion on neonatal outcomes in subsequent pregnancy. Contraception. 2013;87(1):38–44. doi: 10.1016/j.contraception.2012.08.043 23040132

[33] Basso O, Olsen J, Christensen K. Risk of preterm delivery, low birthweight and growth retardation following spontaneous abortion: a registry-based study in Denmark. Int J Epidemiol. 1998 Aug;27(4):642–6. doi: 10.1093/ije/27.4.642 9758119

[34] DaVanzo J, Hale L, Razzaque A, Rahman M. Effects of interpregnancy interval and outcome of the preceding pregnancy on pregnancy outcomes in Matlab. Bangladesh BJOG. 2007;114(9):1079–1087. doi: 10.1111/j.1471-0528.2007.01338.x 17617195 PMC2366022

[35] Regan AK, Gissler M, Magnus MC, Haberg SE, Ball S, Malacova E, et al. Association between interpregnancy interval and adverse birth outcomes in women with a previous stillbirth: an international cohort study. Lancet. 2019;393(10180):1527–1535. doi: 10.1016/S0140-6736(18)32266-9 30827781

[36] MarinovichML, ReganAK, GisslerM, MagnusMC, HåbergSE, MayoJA, et al. Associations between interpregnancy interval and preterm birth by previous preterm birth status in four high-income countries: a cohort study. BJOG 2021 Jun;128(7):1134–1143. doi: 10.1111/1471-0528.16606 33232573

[37] Gisholt MR. Sammen om det vanskelige valget–sykepleie til abortsøkende kvinner: Høgskulen på Vestandet. 2019 [2021 May 03]. Available from: https://www.helsenorge.no/sex-og-samliv/abort/.

[38] Smits LJ, Essed GG. Short interpregnancy intervals and unfavourable pregnancy outcome: role of folate depletion. Lancet. 2001;358(9298):2074–2077. doi: 10.1016/S0140-6736(01)07105-7 11755634

[39] Wilcox AJ, Weinberg CR, O’Connor JF, Baird DD, Schlatterer JP, Canfield RE, et al. Incidence of early loss of pregnancy. N Engl J Med. 1988;319(4):189–194. doi: 10.1056/NEJM198807283190401 3393170

[40] Eskild A, Vatten LJ, Nesheim B-I, Vangen S. The estimated risk of miscarriage should be corrected for induced abortion rates. Acharya G Acta Obstet Gynecol Scand. 2009;88(5):569–574. doi: 10.1080/00016340902814567 19253060

[41] Løkeland M, Bjørge T, Iversen O-E, Akerkar R, Bjørge L. Implementing medical abortion with mifepristone and misoprostol in Norway 1998–2013. Int J Epidemiol. 2016;46(2):643–651. doi: 10.1093/ije/dyw270 28031316 PMC5837406

[42] Bjørge L, Løkeland M, KS. O. Provosert abort. In: Maltau JM, Thornhill HF, Ellstrøm Engh M, editors. Nasjonal veileder I generell gynekologi [National guidelines for general gynaecology]. Oslo2009.

[43] Zhao Y, Zhao Y, Fan K, Jin L. Association of History of Spontaneous or Induced Abortion With Subsequent Risk of Gestational Diabetes. JAMA Netw Open. 2022;5(3):e220944–e. doi: 10.1001/jamanetworkopen.2022.0944 35238932 PMC8895257

[44] Yang H, Wei Y, Gao X, Xu X, Fan L, He J, et al. Risk factors for gestational diabetes mellitus in Chinese women—a prospective study of 16 286 pregnant women in China. Diabet Med. 2009;26(11):1099–104. doi: 10.1111/j.1464-5491.2009.02845.x 19929987

[45] Hutcheon JA, Moskosky S, Ananth CV, Basso O, Briss PA, Ferre CD, et al. Good practices for the design, analysis, and interpretation of observational studies on birth spacing and perinatal health outcomes. Paediatr Perinat Epidemiol. 2019;33(1):O15–o24. Epub 2018/10/13. doi: 10.1111/ppe.12512 30311958 PMC6378590

[46] Silva LM, Coolman M, Steegers EA, Jaddoe VW, Moll HA, Hofman A, et al. Low socioeconomic status is a risk factor for preeclampsia: the Generation R Study. J Hypertens. 2008;26(6):1200–1208. doi: 10.1097/HJH.0b013e3282fcc36e 18475158

[47] Munim S, Maheen H. Association of gestational weight gain and pre-pregnancy body mass index with adverse pregnancy outcome. J Coll Physicians Surg Pak. 2012;22(11):694. doi: 11.2012/JCPSP.694698 23146848

[48] Mohllajee AP, Curtis KM, Morrow B, Marchbanks PA. Pregnancy Intention and Its Relationship to Birth and Maternal Outcomes. Obstet Gynecol. 2007;109(3). doi: 10.1097/01.AOG.0000255666.78427.c5 17329520

