# Supplementary material for: Interpregnancy interval and adverse pregnancy outcomes among pregnancies following miscarriages or induced abortions in Norway (2008–2016): A cohort study
Source: PLoS Med. 2022 Nov 22;19(11):e1004129. doi: 10.1371/journal.pmed.1004129 (PMC9681073; doi:10.1371/journal.pmed.1004129)
Supplement: S1 Table — GDM, gestational diabetes mellitus; LGA, large for gestational age; PTB, preterm birth; SGA, small for gestational age. *Births with nonspontaneous preterm outcomes were excluded when defining spontaneous PTB. (DOCX) [file pmed.1004129.s002.docx]

S1Table. Adverse pregnancy outcomes after miscarriages between
2008 and 2016 in Norway (n=49,058)

| **Outcomes** | **Number (%)** |
| --- | --- |
| **PTB (n= 49,058)** |  |
| No | 46,085 (94.0) |
| Yes | 2,973 (6.0) |
| **Spontaneous PTB (n=47,780)*** |  |
| No | 46,085 (96.5) |
| Yes | 1,695 (3.5) |
| **SGA (n= 49,058)** |  |
| No | 44,404 (90.5) |
| Yes | 4,654 (9.5) |
| **LGA (n= 49,058)** |  |
| No | 44,015 (89.7) |
| Yes | 5,043 (10.3) |
| **Pre-eclampsia (n= 49,058)** |  |
| No | 47.512 (96.8) |
| Yes | 1,546 (3.2) |
| **GDM (n= 49,058)** |  |
| No | 48,984 (95.8) |
| Yes | 2,074 (4.2) |

PTB- preterm birth. SGA- Small-for-gestational age. LGA- Large-for-gestational age.
 GDM- Gestational diabetes mellitus. *Births with non-spontaneous preterm outcomes
were excluded when defining spontaneous PTB.
