# Supplementary material for: Interpregnancy interval and adverse pregnancy outcomes among pregnancies following miscarriages or induced abortions in Norway (2008–2016): A cohort study
Source: PLoS Med. 2022 Nov 22;19(11):e1004129. doi: 10.1371/journal.pmed.1004129 (PMC9681073; doi:10.1371/journal.pmed.1004129)
Supplement: S2 Table — GDM, gestational diabetes mellitus; LGA, large for gestational age; PTB, preterm birth; SGA, small for gestational age. *Births with nonspontaneous preterm outcomes were excluded when defining spontaneous PTB. (DOCX) [file pmed.1004129.s003.docx]

S2 Table. Adverse pregnancy outcomes after induced abortion between
2008 and 2016 in Norway (n=23,707)

| **Outcomes** | **Number (%)** |
| --- | --- |
| **PTB (n=23,707)** |  |
| No | 22,412 (94.5) |
| Yes | 1,295 (5.5) |
| **Spontaneous PTB (n=23,163)*** |  |
| No | 22,412 (96.8) |
| Yes | 751 (3.2) |
| **SGA (n=23,707)** |  |
| No | 21,149 (89.2) |
| Yes | 2,558 (10.8) |
| **LGA (n=23,707)** |  |
| No | 21,598 (91.2) |
| Yes | 2,109 (8.8) |
| **Pre-eclampsia (n=23,707)** |  |
| No | 23,042 (97.2) |
| Yes | 665 (2.8) |
| **GDM (n=23,707)** |  |
| No | 22,955 (96.8) |
| Yes | 752 (3.2) |

PTB- preterm birth. SGA- Small-for-gestational age. LGA- Large-for-gestational age.
GDM- Gestational diabetes mellitus. *Births with non-spontaneous preterm outcomes were
excluded when defining spontaneous PTB.
