# Supplementary material for: Interpregnancy interval and adverse pregnancy outcomes among pregnancies following miscarriages or induced abortions in Norway (2008–2016): A cohort study
Source: PLoS Med. 2022 Nov 22;19(11):e1004129. doi: 10.1371/journal.pmed.1004129 (PMC9681073; doi:10.1371/journal.pmed.1004129)
Supplement: S3 Table — aRR, adjusted relative risk; BMI, body mass index; CI, confidence interval; GDM, gestational diabetes mellitus; IPI, interpregnancy interval; LGA, large for gestational age; PTB, preterm birth; RR, relative risk; SGA, small for gestational age. *Births with nonspontaneous preterm outcomes were excluded when defining spontaneous PTB. **Adjusted for maternal age, gravidity, year of birth, maternal smoking during pregnancy, and prepregnancy BMI at the time of birth after interval. For maternal age and prepregnancy BMI variables, we used restricted cubic splines with 5 knots placed at the 5th, 27.5th, 50th, 72.5th, and 95th percentiles in the study population, which corresponds to 21, 26, 30, 33, and 40 years for maternal age, and 19, 21, 24, 27, and 34 kg/m2 for prepregnancy BMI. (DOCX) [file pmed.1004129.s004.docx]

S3 Table. Sensitivity analysis – Interpregnancy interval after previous miscarriage and risk of adverse pregnancy outcomes with complete information on maternal smoking during pregnancy and pre-pregnancy body mass index for births between 2008 and 2016 in Norway (n =27,747)

| **Outcome** | **IPI** | **Number of cases (%)** | **RR (95% CI)** | **aRR (95% CI)**** | **P-value for aRR** | **E-value for aRR (lower 95% CI)** |
| --- | --- | --- | --- | --- | --- | --- |
| **PTB**  **(n =27,747)** | <3 m | 523(5.5) | 0.89 (0.78, 1.02) | 0.93 (0.81, 1.06) | 0.26 | 1.39 (1) |
|  | 3-5 m | 381 (5.3) | 0.87 (0.75, 0.99) | 0.89 (0.77, 1.02) | 0.09 | 1.50 (1) |
|  | 6-11 m | 346 (6.2) | Ref | Ref |  | Ref |
|  | 12-17 m | 140 (6.6) | 1.06 (0.88, 1.28) | 1.04 (0.86, 1.26) | 9.67 | 1.24 (1) |
|  | 18-23 m | 84 (7.2) | 1.16 (0.93, 1.47) | 1.14 (0.91, 1.44) | 0.25 | 1.21 (1) |
|  | 24-59 m | 136 (6.2) | 1.00 (0.82, 1.21) | 0.99 (0.82, 1.20) | 0.93 | 1.11 (1) |
| **Spontaneous PTB (n=27,025) *** | <3 m | 307 (3.3) | 0.92 (0.77, 1.09) | 0.92 (0.77, 1.10) | 0.36 | 1.39 (1) |
|  | 3-5 m | 204 (3.1) | 0.81 (0.67, 0.98) | 0.82 (0.67, 0.99) | 0.04 | 1.74 (1.11) |
|  | 6-11 m | 197 (3.6) | Ref | Ref |  | Ref |
|  | 12-17 m | 66 (3.2) | 0.89 (0.67, 1.16) | 0.88 (0.67, 1.16) | 0.38 | 1.53 (1) |
|  | 18-23 m | 43 (3.8) | 1.06 (0.77, 1.47) | 1.05 (0.76, 1.45) | 0.77 | 1.21 (1) |
|  | 24-59 m | 71 (3.2) | 0.92 (0.70, 1.20) | 0.91 (0.69, 1.20) | 0.59 | 1.43 (1) |
| **SGA**  **(n =27,025)** | <3 m | 827 (8.5) | 0.85 (0.77, 0.94) | 0.86 (0.78, 0.96) | 0.01 | 1.56 (1.25) |
|  | 3-5 m | 649 (8.8) | 0.88 (0.79, 0.98) | 0.89 (0.80, 0.99) | 0.03 | 1.50 (1.11) |
|  | 6-11 m | 575 (10.0) | Ref | Ref |  | Ref |
|  | 12-17 m | 266 (12.1) | 1.21 (1.06, 1.39) | 1.16 (1.01, 1.34) | 0.03 | 1.64 (1.21) |
|  | 18-23 m | 120 (10.1) | 1.01 (0.84, 1.21) | 0.96 (0.80, 1.16) | 0.68 | 1.16 (1) |
|  | 24-59 m | 242 (11.0) | 1.10 (0.95, 1.26) | 1.00 (0.86, 1.15) | 0.95 | 1.11 (1) |
| **LGA**  **(n =27,747)** | <3 m | 1,010 (10.6) | 1.04 (0.95, 1.15) | 1.07 (0.97, 1.18) | 0.18 | 1.24 (1) |
|  | 3-5 m | 757 (10.6) | 1.04 (0.94, 1.15) | 1.05 (0.95, 1.17) | 0.32 | 1.28 (1) |
|  | 6-11 m | 571 (10.2) | Ref | Ref |  | Ref |
|  | 12-17 m | 215 (10.1) | 0.99 (0.85, 1.14) | 1.00 (0.87, 1.16) | 0.97 | 1.11 (1) |
|  | 18-23 m | 125 (10.8) | 1.06 (0.88, 1.27) | 1.08 (0.90, 1.29) | 0.42 | 1.31 (1) |
|  | 24-59 m | 216 (9.8) | 0.96 (0.83, 1.11) | 1.01 (0.87, 1.17) | 0.91 | 1.11 (1) |
| **Pre-eclampsia**  **(n =27,747)** | <3 m | 307 (3.2) | 0.96 (0.81, 1.15) | 1.00 (0.84, 1.20) | 0.98 | 1.16 (1) |
|  | 3-5 m | 219 (3.1) | 0.91 (0.75, 1.11) | 0.94 (0.78, 1.14) | 0.52 | 1.32 (1) |
|  | 6-11 m | 188 (3.4) | Ref | Ref |  | Ref |
|  | 12-17 m | 88 (4.1) | 1.23 (0.96, 1.57) | 1.21 (0.95, 1.55) | 0.13 | 1.54 (1) |
|  | 18-23 m | 48 (4.1) | 1.23 (0.90, 1.68) | 1.21 (0.89, 1.65) | 0.22 | 1.67 (1) |
|  | 24-59 m | 75 (3.4) | 1.01 (0.78, 1.32) | 0.98 (0.75, 1.32) | 0.87 | 1.11 (1) |
| **GDM**  **(n =27,747)** | <3 m | 372 (3.9) | 0.74 (0.64, 0.86) | 0.84 (0.73, 0.98) | 0.02 | 1.67 (1.29) |
|  | 3-5 m | 333 (4.5) | 0.86 (0.74,1.00) | 0.92 (0.80, 1.07) | 0.26 | 1.39 (1) |
|  | 6-11 m | 296 (5.4) | Ref | Ref |  | Ref |
|  | 12-17 m | 136 (6.4) | 1.20 (0.99, 1.47) | 1.13 (0.93, 1.37) | 0.23 | 1.46 (1) |
|  | 18-23 m | 84 (7.2) | 1.37 (1.12, 1.73) | 1.25 (0.99, 1.57) | 0.06 | 1.86 (1.16) |
|  | 24-59 m | 159 (7.2) | 1.36 (1.13, 1.64) | 1.14 (0.94, 1.37) | 0.18 | 1.67 (1) |

RR- Relative risk. aRR- adjusted relative risk. CI- Confidence interval. IPI - Interpregnancy interval. PTB - Preterm birth. SGA- Small-for-gestational age. LGA- Large-for-gestational age. GDM- Gestational diabetes mellitus. BMI- Body mass index. *Births with non-spontaneous preterm outcomes were excluded when defining spontaneous PTB. **Adjusted for maternal age, gravidity, year of birth, maternal smoking during pregnancy and pre-pregnancy body mass index (BMI) at the time of birth after interval. For maternal age and pre-pregnancy BMI variables, we used restricted cubic splines with 5 knots placed at the 5^th^, 27.5^th^, 50^th^, 72.5^th^ and 95^th^ percentiles in the study population, which corresponds to 21, 26, 30, 33, and 40 years for maternal age; and 19, 21, 24, 27, and 34 kg/m^2^ for pre-pregnancy BMI.
