# Supplementary material for: Interpregnancy interval and adverse pregnancy outcomes among pregnancies following miscarriages or induced abortions in Norway (2008–2016): A cohort study
Source: PLoS Med. 2022 Nov 22;19(11):e1004129. doi: 10.1371/journal.pmed.1004129 (PMC9681073; doi:10.1371/journal.pmed.1004129)
Supplement: S5 Table — aRR, adjusted relative risk; BMI, body mass index; CI, confidence interval; GDM, gestational diabetes mellitus; IPI, interpregnancy interval; LGA, large for gestational age; PTB, preterm birth; RR, relative risk; SGA, small for gestational age. *Births with nonspontaneous preterm outcomes were excluded when defining spontaneous PTB. **Adjusted for maternal age, gravidity, and year of birth at the time of miscarriage (before interval). For maternal age, we used restricted cubic splines with 5 knots placed at the 5th, 27.5th, 50th, 72.5th, and 95th percentiles in the study population, which corresponds to 21, 26, 30, 33, and 40 years for births after a miscarriage. (DOCX) [file pmed.1004129.s006.docx]

S5 Table. Sensitivity analysis – Adjusted relative risk for the association between interpregnancy interval after a miscarriage and adverse pregnancy outcomes adjusted for covariates prior to IPI (n= 49,058).

| **Outcome** | **IPI** | **RR (95% CI)** | **aRR (95% CI)**** | **P-value for aRR** |
| --- | --- | --- | --- | --- |
| **PTB  (n= 49,058)** | <3 m | 0.94 (0.86, 1.04) | 0.97 (0.88, 1.07) | 0.49 |
|  | 3-5 m | 0.91 (0.82, 1.01) | 0.92 (0.83, 1.02) | 0.13 |
|  | 6-11 m | Ref | Ref |  |
|  | 12-17 m | 1.14 (0.99, 1.31) | 1.12 (0.98, 1.29) | 0.10 |
|  | 18-23 m | 1.23 (1.04, 1.45) | 1.23 (1.03, 1.46) | 0.02 |
|  | ≥24 m | 1.10 (0.95, 1.28) | 1.10 (0.94, 1.28) | 0.23 |
| **Spontaneous PTB (n= 47,780)*** | <3 m | 0.98 (0.86, 1.11) | 0.99 (0.87, 1.13) | 0.90 |
|  | 3-5 m | 0.89 (0.78, 1.03) | 0.90 (0.79, 1.04) | 0.16 |
|  | 6-11 m | Ref | Ref |  |
|  | 12-17 m | 1.08 (0.89, 1.31) | 1.06 (0.87, 1.28) | 0.57 |
|  | 18-23 m | 1.25 (0.99, 1.57) | 1.23 (0.97, 1.55) | 0.08 |
|  | ≥24 m | 1.07 (0.88, 1.31) | 1.02 (0.83, 1.25) | 0.83 |
| **SGA  (n= 49,058)** | <3 m | 0.85 (0.79, 0.92) | 0.87 (0.80, 0.93) | 0.00 |
|  | 3-5 m | 0.89 (0.82, 0.97) | 0.90 (0.83, 0.98) | 0.01 |
|  | 6-11 m | Ref | Ref |  |
|  | 12-17 m | 1.14 (1.02, 1.27) | 1.12 (1.01, 1.25) | 0.03 |
|  | 18-23 m | 0.99 (0.85, 1.14) | 0.97 (0.84, 1.12) | 0.69 |
|  | ≥24 m | 1.14 (1.02, 1.27) | 1.07 (0.96, 1.20) | 0.23 |
| **LGA (n= 49,058 )** | <3 m | 1.02 (0.95, 1.10) | 1.02 (0.94, 1.09) | 0.68 |
|  | 3-5 m | 1.00 (0.93, 1.08) | 0.99 (0.92, 1.07) | 0.86 |
|  | 6-11 m | Ref | Ref |  |
|  | 12-17 m | 1.04 (0.93, 1.16) | 1.06 (0.95, 1.18) | 0.30 |
|  | 18-23 m | 0.98 (0.84, 1.13) | 1.00 (0.88, 1.16) | 0.98 |
|  | ≥24 m | 0.91 (0.80, 1.03) | 0.99 (0.88, 1.12) | 0.90 |
| **Pre-eclampsia  (n= 49,058)** | <3 m | 0.91 (0.80, 1.05) | 0.95 (0.83, 1.09) | 0.49 |
|  | 3-5 m | 0.94 (0.81, 1.08) | 0.96 (0.83, 1.11) | 0.61 |
|  | 6-11 m | Ref | Ref |  |
|  | 12-17 m | 1.12 (0.92, 1.37) | 1.09 (0.90, 1.33) | 0.39 |
|  | 18-23 m | 1.12 (0.87, 1.44) | 1.09 (0.85, 1.40) | 0.50 |
|  | ≥24 m | 1.04 (0.84, 1.38) | 0.95 (0.77, 1.18) | 0.67 |
| **GDM  (n= 49,058)** | <3 m | 0.74 (0.66, 0.84) | 0.73 (0.65, 0.83) | 0.00 |
|  | 3-5 m | 0.89 (0.79, 1.00) | 0.87 (0.77, 0.99) | 0.03 |
|  | 6-11 m | Ref | Ref |  |
|  | 12-17 m | 1.29 (1.10, 1.51) | 1.37 (1.17, 1.60) | 0.00 |
|  | 18-23 m | 1.38 (1.14, 1.68) | 1.62 (1.33, 1.96) | 0.001 |
|  | ≥24 m | 1.43 (1.22, 1.67) | 2.12 (1.81, 2.49) | 0.001 |

RR- Relative risk. aRR- adjusted relative risk. CI - Confidence interval. IPI - Interpregnancy interval. PTB - Preterm birth. SGA- Small-for-gestational age. LGA - Large-for-gestational age. GDM- Gestational diabetes mellitus. BMI - Body mass index. *Births with non-spontaneous preterm outcomes were excluded when defining spontaneous PTB. **Adjusted for maternal age, gravidity, year of birth at the time of miscarriage (before interval). For maternal age, we used restricted cubic splines with 5 knots placed at the 5^th^, 27.5^th^, 50^th^, 72.5^th^ and 95^th^ percentiles in the study population, which corresponds to 21, 26, 30, 33, and 40 years for births after a miscarriage.
