# Supplementary material for: Interpregnancy interval and adverse pregnancy outcomes among pregnancies following miscarriages or induced abortions in Norway (2008–2016): A cohort study
Source: PLoS Med. 2022 Nov 22;19(11):e1004129. doi: 10.1371/journal.pmed.1004129 (PMC9681073; doi:10.1371/journal.pmed.1004129)
Supplement: S6 Table — aRR, adjusted relative risk. BMI, body mass index; CI, confidence interval; GDM, gestational diabetes mellitus; IPI, interpregnancy interval; LGA, large for gestational age; PTB, preterm birth; RR, relative risk; SGA, small for gestational age. *Births with nonspontaneous preterm outcomes were excluded when defining spontaneous PTB. **Adjusted for maternal age, gravidity, and year of birth at the time of miscarriage (before interval). For maternal age, we used restricted cubic splines with 5 knots placed at the 5th, 27.5th, 50th, 72.5th, and 95th percentiles in the study population, which corresponds to 18, 22, 25, 29, and 36 for births after an induced abortion. (DOCX) [file pmed.1004129.s007.docx]

S6 Table. Sensitivity analysis – Adjusted relative risk for the association between interpregnancy interval after an induced abortion and adverse pregnancy outcomes adjusted for covariates prior to IPI (n= 23,707)

| **Outcome** | **IPI** | **RR (95% CI)*** | **aRR (95% CI)**** | **P-value for aRR** |
| --- | --- | --- | --- | --- |
| **PTB  (n= 23,707)** | <3 m | 1.20 (0.97, 1.49) | 1.19 (0.96, 1.48) | 0.12 |
|  | 3-5 m | 0.95 (0.78, 1.16) | 0.95 (0.78, 1.16) | 0.62 |
|  | 6-11 m | Ref | Ref |  |
|  | 12-17 m | 0.82 (0.67, 1.00) | 0.82 (0.67, 1.00) | 0.05 |
|  | 18-23 m | 0.94 (0.77, 1.16) | 0.95 (1.77, 1.17) | 0.61 |
|  | ≥24 m | 1.00 (0.85, 1.15) | 0.99 (0.85, 1.16) | 0.92 |
| **Spontaneous PTB (n= 23,163)*** | <3 m | 1.15 (0.86, 1.54) | 1.15 (0.86, 1.54) | 0.36 |
|  | 3-5 m | 1.04 (0.81, 1.34) | 1.04 (0.81, 1.34) | 0.76 |
|  | 6-11 m | Ref | Ref |  |
|  | 12-17 m | 0.62 (0.47, 0.83) | 0.62 (0.47, 0.83) | 0.00 |
|  | 18-23 m | 0.99 (0.76, 1.29) | 0.99 (0.76, 1.30) | 0.97 |
|  | ≥24 m | 0.98 (0.80, 1.19) | 0.97 (0.78, 1.18) | 0.71 |
| **SGA (n= 23,707)** | <3 m | 1.16 (0.98, 1.36) | 1.18 (1.00, 1.39) | 0.05 |
|  | 3-5 m | 1.08 (0.95, 1.24) | 1.10 (0.96, 1.26) | 0.18 |
|  | 6-11 m | Ref | Ref |  |
|  | 12-17 m | 1.12 (0.98, 1.28) | 1.11 (0.97, 1.26) | 0.13 |
|  | 18-23 m | 1.06 (0.92, 1.23) | 1.04 (0.90, 1.20) | 0.62 |
|  | ≥24 m | 1.10 (0.99, 1.23) | 1.02 (0.91, 1.14) | 0.73 |
| **LGA  (n= 23,707)** | <3 m | 0.99 (0.83, 1.18) | 0.97 (0.81, 1.16) | 0.72 |
|  | 3-5 m | 0.84 (0.72, 0.99) | 0.83 (0.71, 0.97) | 0.02 |
|  | 6-11 m | Ref | Ref |  |
|  | 12-17 m | 1.00 (0.87, 1.15) | 1.01 (0.88, 1.17) | 0.85 |
|  | 18-23 m | 0.86 (0.73, 1.01) | 0.86 (0.75, 1.04) | 0.13 |
|  | ≥24 m | 0.95 (0.84, 1.06) | 1.04 (0.93, 1.18) | 0.47 |
| **Pre-eclampsia  (n= 23,707)** | <3 m | 1.13 (0.81, 1.57) | 1.15 (0.83, 1.59) | 0.41 |
|  | 3-5 m | 1.00 (0.76, 1.33) | 1.02 (0.77, 1.36) | 0.88 |
|  | 6-11 m | Ref | Ref |  |
|  | 12-17 m | 0.91 (0.68, 1.20) | 0.90 (0.68, 1.19) | 0.45 |
|  | 18-23 m | 0.92 (0.69, 1.24) | 0.89 (0.66, 1.21) | 0.47 |
|  | ≥24 m | 1.12 (0.91, 1.39) | 1.04 (0.83, 1.30) | 0.73 |
| **GDM  (n= 23,707)** | <3 m | 0.75 (0.52, 1.08) | 0.67 (0.46, 0.96) | 0.03 |
|  | 3-5 m | 0.88 (0.66, 1.16) | 0.82 (0.62, 1.08) | 0.16 |
|  | 6-11 m | Ref | Ref |  |
|  | 12-17 m | 1.02 (0.79, 1.33) | 1.09 (0.84, 1.41) | 0.53 |
|  | 18-23 m | 0.84 (0.62, 1.14) | 0.98 (0.73, 1.32) | 0.90 |
|  | ≥24 m | 1.32 (1.08, 1.62) | 1.95 (1.59, 2.40) | 0.00 |

RR- Relative risk. aRR- adjusted relative risk. CI - Confidence interval. IPI - Interpregnancy interval. PTB - Preterm birth. SGA- Small-for-gestational age. LGA - Large-for-gestational age. GDM- Gestational diabetes mellitus. BMI - Body mass index. *Births with non-spontaneous preterm outcomes were excluded when defining spontaneous PTB. **Adjusted for maternal age, gravidity, year of birth at the time of miscarriage (before interval). For maternal age, we used restricted cubic splines with 5 knots placed at the 5^th^, 27.5^th^, 50^th^, 72.5^th^ and 95^th^ percentiles in the study population, which corresponds to 18, 22, 25, 29, and 36 for births after an induced abortion.
