# Supplementary material for: Interpregnancy interval and adverse pregnancy outcomes among pregnancies following miscarriages or induced abortions in Norway (2008–2016): A cohort study
Source: PLoS Med. 2022 Nov 22;19(11):e1004129. doi: 10.1371/journal.pmed.1004129 (PMC9681073; doi:10.1371/journal.pmed.1004129)
Supplement: S7 Table — BMI, body mass index; CI, confidence interval; GDM, gestational diabetes mellitus; IPI, interpregnancy interval; LGA, large for gestational age; PTB, preterm birth; RR, relative risk; SGA, small for gestational age. *Births with nonspontaneous preterm outcomes were excluded when defining spontaneous PTB. **Adjusted for maternal age, gravidity, and year of birth at the time of birth after interval. For maternal age, we used restricted cubic splines with 5 knots placed at the 5th, 27.5th, 50th, 72.5th, and 95th percentiles in the study population, which corresponds to 21, 26, 30, 33, and 40 years. (DOCX) [file pmed.1004129.s008.docx]

S7 Table. Sensitivity analysis – Interpregnancy interval after previous miscarriage and risk of adverse pregnancy outcomes accounting <6 months of IPI category (n=49,058)

| **Outcome** | **IPI** | **Number of cases (%)** | **RR (95% CI)** | **aRR (95% CI)**** | **P-value for aRR** |
| --- | --- | --- | --- | --- | --- |
| **PTB**  **(n=49,058)** | <6 m | 1,721 (5.7) | 0.93 (0.85, 1.01) | 0.94 (0.86, 1.03) | 0.19 |
|  | 6-11 m | 615 (6.2) | Ref | Ref |  |
|  | 12-17 m | 257 (7.0) | 1.14 (0.99, 1.31) | 1.14 (0.99, 1.31) | 0.08 |
|  | 18-23 m | 150 (7.6) | 1.22 (1.03, 1.46) | 1.23 (1.04, 1.46) | 0.02 |
|  | ≥24 m | 230 (6.8) | 1.10 (0.95, 1.28) | 1.14 (0.98, 1.32) | 0.09 |
| **Spontaneous PTB* (n= 47,780)** | <6 m | 995 (3.4) | 0.94 (0.83, 1.06) | 0.94 (0.83, 1.06) | 0.29 |
|  | 6-11 m | 350 (3.6) | Ref | Ref |  |
|  | 12-17 m | 137 (3.8) | 1.08 (0.89, 1.31) | 1.08 (0.89, 1.31) | 0.43 |
|  | 18-23 m | 86 (4.5) | 1.25 (0.99, 1.57) | 1.26 (1.00, 1.59) | 0.05 |
|  | ≥24 m | 127 (3.9) | 1.07 (0.88, 1.31) | 1.12 (0.92, 1.37) | 0.25 |
| **SGA**  **(n=49,058)** | <6 m | 2,640 (8.8) | 0.87 (0.82, 0.93) | 0.87 (0.81, 0.93) | 0.00 |
|  | 6-11 m | 1,008 (10.1) | Ref | Ref |  |
|  | 12-17 m | 420 (11.5) | 1.14 (1.02, 1.27) | 1.13 (1.02, 1.26) | 0.03 |
|  | 18-23 m | 197 (10.0) | 10.99 (0.85, 1.14) | 0.97 (0.84, 1.12) | 0.70 |
|  | ≥24 m | 389 (11.5) | 1.14 (1.02, 1.27) | 1.09 (0.97, 1.21) | 0.15 |
| **LGA**  **(n=49,058)** | <6 m | 3,121 (10.4) | 1.01 (0.95, 1.08) | 1.01 (0.94, 1.08) | 0.83 |
|  | 6-11 m | 1,021 (10.3) | Ref | Ref |  |
|  | 12-17 m | 390 (10.7) | 1.04 (0.93, 1.16) | 1.06 (0.94, 1.19) | 0.28 |
|  | 18-23 m | 197 (10.0) | 0.98 (0.85, 1.13) | 1.00 (0.86, 1.15) | 0.95 |
|  | ≥24 m | 314 (9.3) | 0.91 (0.80, 1.02) | 0.97 (0.86, 1.09) | 0.63 |
| **Pre-eclampsia**  **(n=49,058)** | <6 m | 903 (3.0) | 0.92 (0.82, 1.06) | 0.94 (0.83, 1.06) | 0.30 |
|  | 6-11 m | 324 (3.4) | Ref | Ref |  |
|  | 12-17 m | 133 (3.6) | 1.12 (0.92, 1.37) | 1.11 (0.91, 1.35) | 0.32 |
|  | 18-23 m | 72 (3.7) | 1.12 (0.87, 1.44) | 1.11 (0.86, 1.42) | 0.44 |
|  | ≥24 m | 114 (3.4) | 1.04 (0.84, 1.28) | 1.01 (0.82, 1.25) | 0.92 |
| **GDM**  **(n=49,058)** | <6 m | 1,079 (3.6) | 0.80 (0.72, 0.89) | 0.89 (0.80, 0.99) | 0.04 |
|  | 6-11 m | 446 (4.4) | Ref | Ref |  |
|  | 12-17 m | 211 (5.8) | 1.29 (1.11, 1.51) | 1.20 (1.02, 1.40) | 0.02 |
|  | 18-23 m | 122 (6.2) | 1.38 (1.17, 1.68) | 1.24 (1.02, 1.50) | 0,03 |
|  | ≥24 m | 216 (6.4) | 1.43 (1.22, 1.67) | 1.14 (0.97, 1.22) | 0.10 |

RR- Relative risk. aRR- adjusted relative risk. CI - Confidence interval. IPI - Interpregnancy interval. PTB - Preterm birth. SGA- Small-for-gestational age. LGA - Large-for-gestational age. GDM- Gestational diabetes mellitus. BMI - Body mass index. *Births with non-spontaneous preterm outcomes were excluded when defining spontaneous PTB. **Adjusted for maternal age, gravidity, year of birth at the time of birth after interval. For maternal age, we used restricted cubic splines with 5 knots placed at the 5^th^, 27.5^th^, 50^th^, 72.5^th^ and 95^th^ percentiles in the study population, which corresponds to 21, 26, 30, 33 and 40 years.
