# Supplementary material for: Interpregnancy interval and adverse pregnancy outcomes among pregnancies following miscarriages or induced abortions in Norway (2008–2016): A cohort study
Source: PLoS Med. 2022 Nov 22;19(11):e1004129. doi: 10.1371/journal.pmed.1004129 (PMC9681073; doi:10.1371/journal.pmed.1004129)
Supplement: S8 Table — aRR, adjusted relative risk; BMI, body mass index; CI, confidence interval; GDM, gestational diabetes mellitus; IPI, interpregnancy interval; LGA, large for gestational age; PTB, preterm birth; RR, relative risk; SGA, small for gestational age. *Births with nonspontaneous preterm outcomes were excluded when defining spontaneous PTB. **Adjusted for maternal age, gravidity, and year of birth at the time of birth after interval. For maternal age, we used restricted cubic splines with 5 knots placed at the 5th, 27.5th, 50th, 72.5th, and 95th percentiles in the study population, which corresponds to 20, 25, 28, 32, and 38 years. (DOCX) [file pmed.1004129.s009.docx]

S8 Table. Sensitivity analysis – Interpregnancy interval after previous induced abortion and risk of adverse pregnancy outcomes accounting <6 months of IPI category (n= 23,707)

| **Outcome** | **IPI** | **Number of cases (%)** | **RR (95% CI)** | **aRR (95% CI)*** | **P-value for aRR** |
| --- | --- | --- | --- | --- | --- |
| **PTB**  **(n= 23,707)** | <6 m | 267(5.8) | 1.04 (0.88, 1.23) | 1.02 (0.86, 1.21) | 0.79 |
|  | 6-11 m | 238 (5.6) | Ref | Ref |  |
|  | 12-17 m | 150 (4.6) | 0.82 (0.67, 1.00) | 0.84 (0.69, 1.02) | 0.08 |
|  | 18-23 m | 134 (5.3) | 0.94 (0.77, 1.16) | 0.97 (0.79, 1.20) | 0.80 |
|  | ≥24 m | 421 (5.6) | 0.99 (0.85, 1.15) | 1.09 (0.94, 1.30) | 0.22 |
| **Spontaneous PTB (n=23,163)** | < 6 m | 164 (3.7) | 1.08 (0.86, 1.35) | 1.06 (0.85, 1.33) | 0.58 |
|  | 6-11 m | 141 (3.4) | Ref | Ref |  |
|  | 12-17 m | 67 (2.1) | 0.62 (0.47, 0.83) | 0.63 (0.47, 0.84) | 0.00 |
|  | 18-23 m | 84 (3.4) | 0.99 (0.76, 1.29) | 1.02 (0.78, 1.33) | 0.90 |
|  | ≥24 m | 295 (3.3) | 0.98 (0.80, 1.19) | 1.06 (0.86, 1.31) | 0.56 |
| **SGA**  **(n=23,707)** | <6 m | 506 (11.1) | 1.11 (0.98, 1.25) | 1.11 (0.99, 1.26) | 0.06 |
|  | 6-11 m | 423 (10.0) | Ref | Ref |  |
|  | 12-17 m | 363 (11.2) | 1.12 (0.98, 1.28) | 1.12 (0.98, 1.28) | 0.09 |
|  | 18-23 m | 269 (10.6) | 1.06 (0.92, 1.23) | 1.07 (0.92, 1.24) | 0.37 |
|  | ≥24 m | 997 (11.0) | 1.10 (0.99, 1.23) | 1.12 (1.00, 1.26) | 0.05 |
| **LGA**  **(n=23,707)** | <6 m | 386 (8.4) | 0.90 (0.78, 1.02) | 0.89 (0.78, 1.01) | 0.08 |
|  | 6-11 m | 400 (9.4) | Ref | Ref |  |
|  | 12-17 m | 307 (9.4) | 1.00 (0.87, 1.15) | 1.00 (0.87, 1.15) | 0.99 |
|  | 18-23 m | 205 (8.1) | 0.86 (0.73, 1.01) | 0.86 (0.73, 1.01) | 0.06 |
|  | ≥24 m | 811 (8.9) | 0.95 (0.84, 1.06) | 0.95 (0.84, 1.07) | 0.41 |
| **Pre-eclampsia**  **(n=23,707)** | <6 m | 130 (2.8) | 1.05 (0.82, 1.34) | 1.07 (0.83, 1.36) | 0.62 |
|  | 6-11 m | 115 (2.7) | Ref | Ref |  |
|  | 12-17 m | 80 (2.5) | 0.91 (0.68, 1.20) | 0.91 (0.68, 1.20) | 0.49 |
|  | 18-23 m | 63 (2.5) | 0.92 (0.68, 1.24) | 0.91 (0.67, 1.23) | 0.53 |
|  | ≥24 m | 277 (3.1) | 1.12 (0.91, 1.39) | 1.09 (0.87, 1.37) | 0.46 |
| **GDM**  **(n=23,707)** | <6 m | 112 (2.5) | 0.83 (0.65, 1.07) | 0.83 (0.65, 1.07) | 0.16 |
|  | 6-11 m | 125 (3.0) | Ref | Ref |  |
|  | 12-17 m | 98 (3.0) | 1.02 (0.79, 1.33) | 0.97 (0.75, 1.25) | 0.81 |
|  | 18-23 m | 63 (2.5) | 0.84 (0.62, 1.14) | 0.79 (0.59, 1.06) | 0.12 |
|  | ≥24 m | 354 (3.9) | 1.32 (1.08, 1.62) | 1.06 (0.85, 1.28) | 0.72 |

RR- Relative risk. aRR- adjusted relative risk. CI - Confidence interval. IPI - Interpregnancy interval. PTB - Preterm birth. SGA- Small-for-gestational age. LGA - Large-for-gestational age. GDM- Gestational diabetes mellitus. BMI - Body mass index. *Births with non-spontaneous preterm outcomes were excluded when defining spontaneous PTB. *Adjusted for maternal age, gravidity, year of birth at the time of birth after interval. For maternal age, we used restricted cubic splines with 5 knots placed at the 5^th^, 27.5^th^, 50^th^, 72.5^th^ and 95^th^ percentiles in the study population, which corresponds to 20, 25, 28, 32 and 38 years.
