# Supplementary material for: Interpregnancy interval and adverse pregnancy outcomes among pregnancies following miscarriages or induced abortions in Norway (2008–2016): A cohort study
Source: PLoS Med. 2022 Nov 22;19(11):e1004129. doi: 10.1371/journal.pmed.1004129 (PMC9681073; doi:10.1371/journal.pmed.1004129)
Supplement: S9 Table — aRR, adjusted relative risk; BMI, body mass index; CI, confidence interval; GDM, gestational diabetes mellitus; IPI, interpregnancy interval; LGA, large for gestational age; PTB, preterm birth; RR, relative risk; SGA, small for gestational age. *Births with nonspontaneous preterm outcomes were excluded when defining spontaneous PTB. *Adjusted for maternal age, gravidity, and year of birth at the time of birth after interval. For maternal age, we used restricted cubic splines with 5 knots placed at the 5th, 27.5th, 50th, 72.5th, and 95th percentiles in the study population, which corresponds to 21, 26, 30, 33, and 40 years. **E-values for unmeasured confounding for the association between IPI after miscarriage and induced abortion and adverse pregnancy outcomes. (DOCX) [file pmed.1004129.s010.docx]

S9 Table. Sensitivity analysis – Interpregnancy interval after previous miscarriages and risk of adverse pregnancy outcomes among births from women with only one miscarriage in the cohort (n= 47,411)

| **Outcome** | | **IPI** | **Number of  cases (%)** | **RR (95% CI)** | **aRR (95% CI)**** | **P-value for aRR** |
| --- | --- | --- | --- | --- | --- | --- |
| **PTB**  **(n=47,411)** | <3 m | 965 (5.8) | 0.94 (0.86, 1.04) | 0.96 (0.87, 1.06) | 0.42 |  |
|  | 3-5 m | 692 (5.6) | 0.91 (0.82, 1.01) | 0.92 (0.83, 1.03) | 0.14 |  |
|  | 6-11 m | 593 (6.2) | Ref | Ref |  |  |
|  | 12-17 m | 246 (6.9) | 1.12 (0.97, 1.30) | 1.12 (0.97, 1.29) | 0.12 |  |
|  | 18-23 m | 149 (7.7) | 1.26 (1.06, 1.50) | 1.26 (1.06, 1.49) | 0.01 |  |
|  | ≥24 m | 226 (6.8) | 1.10 (0.95, 1.28) | 1.14 (0.98, 1.30) | 0.09 |  |
| **Spontaneous PTB*  (n= 46,178)** | < 3 m | 569 (3.5) | 0.97 (0.86, 1.11) | 0.97 (0.85, 1.10) | 0.61 |  |
|  | 3-5 m | 385 (3.2) | 0.89 (0.77, 1.02) | 0.89 (0.77, 1.02) | 0.10 |  |
|  | 6-11 m | 339 (3.6) | Ref | Ref |  |  |
|  | 12-17 m | 133 (3.9) | 1.07 (0.88, 1.30) | 1.08 (0.88, 1.31) | 0.47 |  |
|  | 18-23 m | 86 (4.6) | 1.28 (1.02, 1.61) | 1.29 (1.02, 1.63) | 0.03 |  |
|  | ≥24 m | 126 (3.9) | 1.08 (0.88, 1.32) | 1.13 (0.92, 1.38) | 0.23 |  |
| **SGA**  **(n=47,411)** | <3 m | 1,443 (8.7) | 0.85 (0.79, 0.92) | 0.85 (0.79, 0.92) | 0.00 |  |
|  | 3-5 m | 1,114 (9.0) | 0.89 (0.82, 0.97) | 0.88 (0.81, 0.96) | 0.00 |  |
|  | 6-11 m | 983 (10.1) | Ref | Ref |  |  |
|  | 12-17 m | 415 (11.7) | 1.14 (1.03, 1.27) | 1.14 (1.02, 1.27) | 0.02 |  |
|  | 18-23 m | 193 (10.0) | 0.98 (0.85, 1.14) | 0.97 (0.84, 1.12) | 0.66 |  |
|  | ≥24 m | 386 (11.6) | 1.13 (1.02, 1.27) | 1.08 (0.97, 1.21) | 0.16 |  |
| **LGA**  **(n=47,411)** | <3 m | 1,724 (10.4) | 1.04 (0.96, 1.12) | 1.05 (0.98, 1.13) | 0.18 |  |
|  | 3-5 m | 1,244 (10.0) | 1.01 (0.93, 1.09) | 1.02 (0.94, 1.10) | 0.84 |  |
|  | 6-11 m | 965 (10.0) | Ref | Ref |  |  |
|  | 12-17 m | 376 (10.6) | 1.06 (0.94, 1.18) | 1.06 (0.95, 1.19) | 0.29 |  |
|  | 18-23 m | 189 (9.8) | 0.98 (0.85, 1.14) | 1.00 (0.86, 1.16) | 0.99 |  |
|  | ≥24 m | 308 (9.2) | 0.92 (0.82, 1.04) | 0.97 (0.86, 1.10) | 0.64 |  |
| **Pre-eclampsia**  **(n=47,411)** | <3 m | 495 (3.0) | 0.91 (0.79, 1.04) | 0.92 (0.80, 1.06) | 0.23 |  |
|  | 3-5 m | 381 (3.1) | 0.94 (0.81, 1.09) | 0.95 (0.82, 1.10) | 0.46 |  |
|  | 6-11 m | 317 (3.3) | Ref | Ref |  |  |
|  | 12-17 m | 131 (3.7) | 1.12 (0.92, 1.37) | 1.11 (0.91, 1.35) | 0.32 |  |
|  | 18-23 m | 72 (3.7) | 1.14 (0.89, 1.46) | 1.12 (0.87, 1.44) | 0.37 |  |
|  | ≥24 m | 113 (3.4) | 1.03 (0.83, 1.27) | 1.01 (0.81, 1.25) | 0.94 |  |
| **GDM**  **(n=47,411)** | <3 m | 542 (3.3) | 0.74 (0.65, 0.83) | 0.84 (0.74, 0.95) | 0.01 |  |
|  | 3-5 m | 483 (3.9) | 0.88 (0.78, 1.00) | 0.95 (0.84, 1.08) | 0.45 |  |
|  | 6-11 m | 427 (4.4) | Ref | Ref |  |  |
|  | 12-17 m | 199 (5.6) | 1.26 (1.07, 1.49) | 1.16 (0.99, 1.37) | 0.07 |  |
|  | 18-23 m | 116 (6.0) | 1.36 (1.12, 1.66) | 1.21 (1.00, 1.48) | 0.05 |  |
|  | ≥24 m | 214 (6.4) | 1.45 (1.23, 1.70) | 1.14 (0.97, 1.34) | 0.10 |  |

RR- Relative risk. aRR- adjusted relative risk. CI - Confidence interval. IPI - Interpregnancy interval. PTB - Preterm birth. SGA- Small-for-gestational age. LGA - Large-for-gestational age. GDM- Gestational diabetes mellitus. BMI - Body mass index. *Births with non-spontaneous preterm outcomes were excluded when defining spontaneous PTB. *Adjusted for maternal age, gravidity, year of birth at the time of birth after interval. For maternal age, we used restricted cubic splines with 5 knots placed at the 5^th^, 27.5^th^, 50^th^, 72.5^th^ and 95^th^ percentiles in the study population, which corresponds to 21, 26, 30, 33 and 40 years.
