# Supplementary material for: Interpregnancy interval and adverse pregnancy outcomes among pregnancies following miscarriages or induced abortions in Norway (2008–2016): A cohort study
Source: PLoS Med. 2022 Nov 22;19(11):e1004129. doi: 10.1371/journal.pmed.1004129 (PMC9681073; doi:10.1371/journal.pmed.1004129)
Supplement: S10 Table — aRR, adjusted relative risk; BMI, body mass index; CI, confidence interval; GDM, gestational diabetes mellitus; IPI, interpregnancy interval; LGA, large for gestational age; PTB, preterm birth; RR, relative risk; SGA, small for gestational age. *Births with nonspontaneous preterm outcomes were excluded when defining spontaneous PTB. **Adjusted for maternal age, gravidity, and year of birth at the time of birth after interval. For maternal age, we used restricted cubic splines with 5 knots placed at the 5th, 27.5th, 50th, 72.5th, and 95th percentiles in the study population, which corresponds to 20, 25, 28, 32, and 38 years. **E-values for unmeasured confounding for the association between IPI after miscarriage and induced abortion and adverse pregnancy outcomes. (DOCX) [file pmed.1004129.s011.docx]

S10 Table. Sensitivity analysis – Interpregnancy interval after previous induced abortion and risk of adverse pregnancy outcomes among births from women with only one induced abortion in the cohort (n= 23,185)

| **Outcome** | **IPI** | **Number of cases (%)** | **RR (95% CI)** | **aRR (95% CI)**** | **P-value for aRR** |
| --- | --- | --- | --- | --- | --- |
| **PTB  (n=23,185**) | <3 m | 105 (6.7) | 1.21 (0.97, 1.52) | 1.18 (0.95, 1.48) | 0.14 |
|  | 3-5 m | 152 (5.3) | 0.96 (0.79, 1.17) | 0.95 (0.78, 1.16) | 0.61 |
|  | 6-11 m | 228 (5.5) | Ref | Ref |  |
|  | 12-17 m | 144 (4.6) | 0.83 (0.67, 1.01) | 0.84 (0.69, 1.03) | 0.10 |
|  | 18-23 m | 130 (5.2) | 0.95 (0.77, 1.17) | 0.98 (0.80, 1.21) | 0.86 |
|  | ≥24 m | 498 (5.5) | 1.00 (0.86, 1.17) | 1.12 (0.95, 1.32) | 0.16 |
| **Spontaneous PTB** **(n=22,657)*** | <3 m | 58 (3.8) | 1.16 (0.86, 1.57) | 1.14 (0.84, 1.55) | 0.40 |
|  | 3-5 m | 100 (3.6) | 1.08 (0.84, 1.39) | 1.07 (0.83, 1.38) | 0.61 |
|  | 6-11 m | 133 (3.3) | Ref | Ref |  |
|  | 12-17 m | 65 (2.1) | 0.64 (0.48, 0.86) | 0.65 (0.49, 0.87) | 0.00 |
|  | 18-23 m | 80 (3.3) | 1.00 (0.76, 131) | 1.02 (0.78, 1.35) | 0.86 |
|  | ≥24 m | 293 (3.3) | 1.01 (0.83, 1.24) | 1.11 (0.90, 1.38) | 0.33 |
| **SGA  (n=23,185)** | <3 m | 183 (11.7) | 1.16 (0.98, 1.37) | 1.16 (0.99, 1.37) | 0.06 |
|  | 3-5 m | 313 (10.9) | 1.09 (0.95, 1.25) | 1.09 (0.95, 1.25) | 0.21 |
|  | 6-11 m | 415 (10.1) | Ref | Ref |  |
|  | 12-17 m | 355 (11.2) | 1.12 (0.98, 1.28) | 1.12 (0.98, 1.28) | 0.09 |
|  | 18-23 m | 266 (10.7) | 1.07 (0.92, 1.23) | 1.07 (0.93, 1.24) | 0.36 |
|  | ≥24 m | 987 (11.0) | 1.09 (0.98, 1.22) | 1.12 (1.00, 1.26) | 0.08 |
| **LGA \| (n=23,185)** | <3 m | 147 (9.4) | 1.01 (0.85, 1.17) | 01.00 (0.84, 1.20) | 0.98 |
|  | 3-5 m | 219 (7.7) | 0.83 (0.71, 0.97) | 0.82 (0.70, 0.96) | 0.02 |
|  | 6-11 m | 382 (9.3) | Ref | Ref |  |
|  | 12-17 m | 291 (9.2) | 1.00 (0.86, 1.15) | 0.99 (0.86, 1.15) | 0.94 |
|  | 18-23 m | 197 7.9) | 0.86 (0.73, 1.01) | 0.86 (0.73, 1.01) | 0.07 |
|  | ≥24 m | 800 (8.9) | 0.96 (0.86, 1.08) | 0.97 (0.86, 1.10) | 0.63 |
| **Pre-eclampsia** **(n=23,185)** | <3 m | 50 (3.2) | 1.13 (0.81, 1.57) | 1.21 (0.87, 1.68) | 0.26 |
|  | 3-5 m | 80 (2.8) | 1.00 (0.76, 1.33) | 1.06 (0.80, 1.40) | 0.71 |
|  | 6-11 m | 111 (2.7) | Ref | Ref |  |
|  | 12-17 m | 76 (2.4) | 0.91 (0.68, 1.20) | 0.89 (0.67, 1.19) | 0.44 |
|  | 18-23 m | 62 (2.5) | 0.92 (0.68, 1.24) | 0.92 (0.68, 1.25) | 0.60 |
|  | ≥24 m | 274 (3.1) | 1.12 (0.91, 1.39) | 1.10 (0.87, 1.38) | 0.43 |
| **GDM (n=23,185)** | <3 m | 36 (2.3) | 0.77 (0.53, 1.11) | 0.78 (0.54, 1.12) | 0.17 |
|  | 3-5 m | 75 (2.6) | 0.88 (0.66, 1.17) | 0.88 (0.66, 1.17) | 0.66 |
|  | 6-11 m | 123 (3.0) | Ref | Ref |  |
|  | 12-17 m | 94 (3.0) | 1.00 (0.77, 1.30) | 0.94 (0.72, 1.23) | 0.66 |
|  | 18-23 m | 59 (2.4) | 0.80 (0.59, 1.08) | 0.74 (0.55, 1.1) | 0.06 |
|  | ≥24 m | 350 (3.9) | 1.31 (1.07, 1.60) | 1.02 (0.83, 1.25) | 0.87 |

RR- Relative risk. aRR- adjusted relative risk. CI - Confidence interval. IPI - Interpregnancy interval. PTB - Preterm birth. SGA- Small-for-gestational age. LGA - Large-for-gestational age. GDM- Gestational diabetes mellitus. BMI - Body mass index. *Births with non-spontaneous preterm outcomes were excluded when defining spontaneous PTB. **Adjusted for maternal age, gravidity, year of birth at the time of birth after interval. For maternal age, we used restricted cubic splines with 5 knots placed at the 5^th^, 27.5^th^, 50^th^, 72.5^th^ and 95^th^ percentiles in the study population, which corresponds to 20, 25, 28, 32, and 38 years.
