# Supplementary material for: Interpregnancy interval and adverse pregnancy outcomes among pregnancies following miscarriages or induced abortions in Norway (2008–2016): A cohort study
Source: PLoS Med. 2022 Nov 22;19(11):e1004129. doi: 10.1371/journal.pmed.1004129 (PMC9681073; doi:10.1371/journal.pmed.1004129)
Supplement: Attachment — Submitted filename: review_PlosMed.docx [file pmed.1004129.s012.docx]

review

general:

This register-based study offers generally valuable information and study questions concern great number of fertile aged women. The findings of the study are also valuable to the health care providers councelling women for future reproduction after miscarriage/induced aboriton. The data is retrieved from the national population-based registers and offers a good insight to the study question.

Abstract:

Authors should describe more precisely what are the “adverse outcomes” they aim to study and what have they been focusing on.

Introduction:

In the introduction, the authors should give more information why they choose to have pre-eclamsia and gestational dieabets in the study. It seems to be a kind of spin-off in the study, and no explanation why to study association of IPI with PE/GDM from the literature has been presented . The aims are a bit unclear and pre-eclamsia and GDM has not been mentioned as the aim. in fact, I would suggest to leave out PE and GDM form the study.

Methods:

The major problem in the setting of this study is that the authors have included all the women during the study period, thus some women may appear in the data various times causing bias. The authors should reanalyze the study population by including each woman just once in the analyses. Also, the data concerning method of treatment (medical/surgical) as well as the duration of gestation in miscarriage/induced abortion would improve the study, as those factors are known to affect risks in pregnancy (for example multiple surgical abortions increase the risk of preterm birth).

Results:

As there are very few women in the IPI group > 24 months, I suggest to combine >24 months and > 60 months. Why not to have smoking in the model shown in the paper, if it does not change the results? Smoking is well known risk factor for SGA for example, and should be in the primary model.

Discussion:

Please see my previous comments on PE and GDM.
