# Supplementary material for: Interpregnancy interval and adverse pregnancy outcomes among pregnancies following miscarriages or induced abortions in Norway (2008–2016): A cohort study
Source: PLoS Med. 2022 Nov 22;19(11):e1004129. doi: 10.1371/journal.pmed.1004129 (PMC9681073; doi:10.1371/journal.pmed.1004129)
Supplement: Attachment — Submitted filename: Point by point response_R2.docx [file pmed.1004129.s013.docx]

**Point-by-point responses to the editor and reviewers**

**Comments from Editor**

General

**Comment #1.** Please revise your title to include the study design in the subtitle such as: “Interpregnancy interval and adverse pregnancy outcomes among pregnancies following miscarriages and induced abortions: a cohort study”

**Response #1.** We have revised the title as suggested. For more clarity, we have also included the study region and period. The new title reads as:

*“Interpregnancy interval and adverse pregnancy outcomes among pregnancies following miscarriages or induced abortions in Norway (2008-2016): a cohort study”*

**Comment #2.** As the data are not freely available, please describe briefly the ethical, legal, or contractual restriction that prevents you from sharing it. Please also include an appropriate contact (web or email address) for inquiries (this cannot be a study author).

**Response #2.** We have now updated the data availability information as “Study data are available on application. Information on how to apply and access data can be found from <https://helsedata.no/>. Data access is subject to compulsory ethics and governance approvals.”

**Comment #3.** Thank you for including an author summary we would suggest emphasizing as a final point in “what do these findings mean” that the results may prompt a review of guidelines, as we understand it

**Response #3.** As suggested we have expanded ‘what do these findings mean’ by including two bullet points.

Author summary in page 4 lines 81-84:

- *In combination with previous research, our results are re-assuring for families who attempt pregnancy soon after a miscarriage or induced abortion.*
- *These findings motivate a revision of the current international guidelines for birth spacing after a miscarriage or an induced abortion.*

**Comment #4**. There are a number of typographical and grammatical errors throughout as detailed below and highlighted by the reviewers. Please check through the manuscript carefully for these errors.

**Response #4.** We have carefully reviewed the manuscript for typographical and grammatical errors.

**ABSTRACT** METHODS and FINDINGS

**Comment #5.** Please include the main outcome measures and define any abbreviations used. Please quantify the main results with p values as well as 95% CIs. Please include the absolute risk(s) of relevant outcomes, not just relative risks (an example for absolute risk inclusion in the abstract can be found here: PMID: 28399126).

**Response #5.** Thank you for providing the reference. and we have restructured our abstract to describe the main outcome measures in the background section of the abstract as: ”… *the risk of adverse pregnancy outcomes — preterm birth (PTB), spontaneous PTB, small for gestational age (SGA) birth, large for gestational age (LGA) birth, preeclampsia, and gestational diabetes mellitus (GDM)*” (Please see Page 2, lines 15-17). We have also defined all abbreviations included in the paper. We have also included absolute risk for relevant outcomes and p-values in the revised version.

Abstract page 2-3, lines 27-41

*“Compared to births with an IPI of 6-11 months after miscarriages (10.1%), there were lower risks of small for gestational age (SGA) births among births with an IPI of <3 months (8.6%) (aRR 0.85, 95% CI: 0.79, 0.92, p-value=0.00) and 3-5 months (9.0%) (aRR 0.90, 95% CI: 0.83, 0.97, p-value=0.01). An IPI of <3 months after a miscarriage (3.3%) was also associated with lower risk of GDM (aRR 0.84, 95 % CI: 0.75, 0.96, p-value=0.01) as compared to an IPI of 6-11 months (4.5%). For births following a previous induced abortion, an IPI <3 months (11.5%) was associated with a non-significant but increased risk of SGA (aRR 1.16, 95 % CI: 0.99, 1.36, p-value=0.07) as compared to an IPI of 6-11 months (10.0%), while the risk of LGA was lower among those with an IPI 3-5 months (8.0%) (aRR 0.84, 95 % CI: 0.72, 0.98, p-value =0.03) compared to an IPI of 6-11 months (9.4%). There was no observed association between adverse pregnancy outcomes with an IPI >12 months after either a miscarriage or induced abortion (p-value>0.05), with the exception of an increased risk of GDM among women with an IPI of 12-17 months (5.8%) (aRR 1.20, 95 % CI: 1.02, 1.40, p-value=0.02), 18-23 months (6.2%) (aRR 1.24, 95% CI: 1.02, 1.50, p-value=0.03), and ≥24 months (6.4%) (aRR 1.14, 95% CI: 0.97, 1.34, p-value=0.10) compared to an IPI of 6-11 months (4.5%) after a miscarriage.”*

**Comment #6.** Please include the important dependent variables that are adjusted for in the analyses.

**Response #6.** We have revised the abstract by including descriptions for variables adjusted in the analysis.

Abstract page 2 lines 24-27:

*“In the adjusted model, we included maternal age, gravidity, year of birth measured at the time of the index (after interval) births. In a sensitivity analysis, we further adjusted for smoking during pregnancy and pre-pregnancy body mass index”*

**Comment #7.** In the last sentence of the Abstract Methods and Findings section, please describe the main limitation(s) of the study's methodology.

**Response #7.** As suggested, we have included a statement describing the main limitation of the study presented as follows:

Abstract page 2 lines 41-42

*“The main limitation of this study was that we did not have information on potential confounders such as pregnancy intention and health seeking behaviour.”*

MAIN MANUSCRIPT METHODS AND RESULTS

**Comment #8.** Participants and exclusions – line 3 please check grammar … “we left with” suggest adding “were”

**Response #8.** Thank you for pointing out this error. It is now corrected.

**Comment #9.** Please provide p values as well as 95% CIs, where a p value is given please also specify the statistical test used to determine it

**Response #9.** We have now included p-values along with the effect size measures both in the in the abstract and main text. We have also included one more column in the tables showing p-values for adjusted relative risk measures. Please see abstract (page 2), results sections (page 13 & 15), and Table 3 & 4.

TABLES

**Comment #10.** Please define the numbers in brackets in table 1 [i.e. 384 (0.8)] ?median Please define the numbers in brackets in table 2 [i.e 47,308 (94.0) ? percentage. Please provide the unadjusted comparisons as well as the adjusted comparisons in Table S3.

**Response #10.** We have now revised our result presentation in Table 1 (now Tables 1 & 2) and Table 2 (now S1 & S2 Tables), updating the column title as “Number (%)”. As requested, we have now included unadjusted comparison for results in Table S3 (now S5 & S6 Tables).

FIGURES

**Comment #11.** Please provide titles and legends for each individual table and figures in the Supporting Information.

**Response #11.** As requested, we have now provided provide titles and legends for each individual table and figures in the Supporting Information. Please see Supporting Information in pages 28-31, lines 549-639.

DISCUSSION

**Comment #12.** Some sentences throughout might benefit from shortening and re-wording to improve clarity and readability for example, in the Strengths and limitations of the study – the sentence on page 19 “…information were only available for pregnancies ending in the birth registry…” is a little confusing

**Response #12.** We have now carefully revised the manuscript for language clarity. For example, the above text has been updated in page 21, lines 363-366 and the full statement reads as follows:

“*However, since smoking and pre-pregnancy BMI information were only available for pregnancies registered in the birth registry, we were not able to adjust for these variables at the time of miscarriage or induced abortion.”*

**Reviewer #1**

**Comment #1.** I confine my remarks to statistical aspects of this paper. The general approach is fine, and I found some aspects commendable, but I do have some issues to resolve before I can recommend publication

**Response #1.** Thank you. Our responses to your comments are provided below.

**Comment #2.** NOTE: Line numbers would have made the review process easier

**Response #2.** We apologize for this oversight. Line numbers have been added in the revised submission.

**Comment #3.** p. 2 "Log binomial" isn't the usual term. I would use "logistic", but this is not a big deal.

**Response #3.** We apologize for not being entirely clear in our description of the model we used. The log binomial is different from logistic model, in that the former uses a log link function while the latter uses a logit link. Log binomial model produce estimate of relative risk instead of odds ratios, and are more appropriate for cohort studies such as ours.

**Comment #4.** Not a stat issue, but SGA and other acronyms should be spelled out at first use.

**Response #4.** Thank you for pointing this out. We have ensured that all abbreviations are defined at first-time use.

**Comment #5.** p. 5 Where possible, please give the effect size estimates from the various studies you report on. The fact that some studies found a significant effect and others did not is not enough. See Andrew Gelman's paper "The difference between significant and nonsignificant is not, itself, significant". Also, where you say "no increased risk" please insert "significant" (or, if they found a decrease, say that).

**Response #5.** Thank you for the reference. As requested, we have provided effect sizes for the studies we reference in the paper. See the following revised statements as an example.

Introduction section page 5, lines 96-107

*“The study from Latin America did not distinguish between miscarriages and induced abortions, which is likely to have resulted in substantial heterogeneity in the underlying risk profile. Furthermore, when large cohort studies from Scotland (OR 0.89, 95% CI: 0.81, 0.98 for PTB; OR 0.84, 95% CI: 0.71, 0.89 for LBW; and OR 1.18, 95% CI: 0.82, 1.23 for preeclampsia) and California (OR 0.87, 95% CI: 0.81, 0.94 for PTB) subsequently evaluated this research question, they observed no increased risk of adverse pregnancy outcomes in births that followed a short IPI (< 6 months) after a miscarriage [10, 11]. However, an increased risk of adverse pregnancy outcomes in pregnancies that followed a short (<6 months) IPI after induced abortions was observed in Finland (OR 1.44, 95% CI: 1.10, 1.88 for PTB) [12]. Studies investigating risks of maternal complications after long IPI following a live birth indicated increased risks of gestational diabetes mellitus in (GDM) in Canada (OR 1.66, 95% CI: 1.39, 2.00) and preeclampsia in Australia (OR 1.28, 95% CI: 1.17, 1.41) [13, 14]. ”*

We strongly prefer to avoid using the term “statistical significance”. As most statisticians agree that the commonly used 5% threshold for “statistical significance” is arbitrary, it is more important to emphasize the magnitude of the effect estimates and the width of plausible values reflected in the confidence interval. We hope that the reviewer can appreciate these nuances.

**Comment #6.1.** p. 7 Do not categorize IPI. Doing so increases type I and type II error and introduces a kind of magical thinking. that something amazing happens at the cutpoints. See my article <https://medium.com/@peterflom/what-happens-when-we-categorize-an-independent-variable-in-regression-77d4c5862b6c> (the article is about linear regression, but applies to logistic as well). If you wish to report results by category, for comparison purposes, that's OK, but the analysis should use IPI as a number, and maybe use splines to look at nonlinearity. Splines could even find that the categories are bad ones.

**Comment #6.2.** p. 8 Same issue for age and BMI as for IPI. BMI could easily have nonlinear effects.

**Response #6.1 & 6.2.** We thank the reviewer for this suggestion and for sharing the reference for guidance. However, in light of our research question that aimed to challenge current international recommendation for woman to wait at least 6 months after miscarriages or induced abortion, we create discrete segments of IPI will facilitate easier understanding and comparison with the WHO recommendations and similar studies. [1, 6, 7]. For our categorisation, we followed standard cut-off points widely used in the IPI literature including publications on the topic from our team (PMID: 35596254, 34857549, 30827781). However, we further categorised <6 months of IPI into <3, 3-5 months of IPI for a detailed examination of short IPI although we have also conducted additional sensitivity analysis to compare our results with studies considering <6 months in their short IPI categories. However, due to smaller cell counts in the last category (i.e., >60 months of IPI) and suggestions from Reviewer #2, we put the last two categories together as > 24 months of IPI. We have provided the categorisations with citations in the methods section.

Methods page 8, lines 161-162 & 194-197, respectively:

*“We categorised IPI into six periods: <3, 3-5, 6-11, 12-17, 18-23, and ≥24 months, with 6-11 months as the reference category, consistent with the WHO guidelines and literature [1, 6, 7].”*

*“Since IPI categorisation that consider <6 months in the short IPI category is widely considered in the literature and for our results to be compared with the WHO recommendation, we conducted additional analysis combining the first two IPI categories (<3 months and 3-5 months).”*

For maternal age and pre pregnancy BMI in our adjustment variables, as suggested, we have used restricted cubic splines for maternal age and pre pregnancy BMI and we have described it in the methods section. For our knot placement, we followed Harrell recommendation to apply 5 knots as also discussed on the online article suggested by our reviewer. We have described the restricted cubic splines placement for maternal age and pre pregnancy BMI.

Methods section in page 8 lines 177-178:

*“… for maternal age using restricted cubic splines with 5 knots placed at the 5^th^, 27.5^th^, 50^th^, 72.5^th^ and 95^th^ percentiles in the study population,[8] which corresponds to 22, 28, 31, 35, and 40 years for births after a miscarriage study population and 20, 25, 28, 32, and 38 years for births after induced abortion study population.”*

Methods section page 9 lines 188-190:

*“… for pre‐pregnancy body mass index (BMI) using restricted cubic splines placed at 5 knots placed at the 5^th^, 27.5^th^, 50^th^, 72.5^th^ and 95^th^ percentiles in the study population, which corresponds to 19, 21, 24, 27, and 34 kg/m2 for births after a miscarriage study population and 18, 21, 23, 36, and 33 kg/m2 years for births after induced abortion study population”*

**Comment #7.** Congratulations for doing some sensitivity analyses. Not enough studies include them

**Response #7.** Thank you

**Comment #8.** I also commend the authors for not using stepwise or other automatic variable selection methods. They are deeply flawed.

**Response #8.** Thank you

**Comment #9.** How were the reference categories for the categorized continuous variables selected? I don't like categorization (see above) but, if you do categorize, there should be some stated reasons for choosing a reference category. The most usual choices are either the most common category or an extreme category. For IPI, both of those would suggest using < 3 month. Also, table 3 shows another problem with categorization - some of the cells are small sized, and the sizes vary a lot, so, the same OR can be significant in one category and not another.

**Response #9.** As we have described above, given our primary research question to challenge the current WHO recommendation and for us to compare with similar studies we chose to use 6-11 months of IPI to be our comparison group. See our response for **Comment #6.** For the adjustment variables, we have chosen the extreme categories.

**Comment #10**. Finally, figure 2 seems to be based on using IPI as a continuous measure. Both of these show very nonlinear relationships. This makes use of splines for IPI more important.

**Response #10.** In Figure 2, we would like to note that we presented the distribution of IPI for those births after a miscarriage and induced abortion separately. This figure did not show the relationship between IPI and any of the pregnancy outcomes in the subsequent birth evaluated in the study.

**Reviewer #2**

General:

**Comment #1.** This register-based study offers generally valuable information and study questions concern great number of fertile aged women. The findings of the study are also valuable to the health care providers counselling women for future reproduction after miscarriage/induced abortion. The data is retrieved from the national population-based registers and offers a good insight to the study question.

**Response #1.** Thank you

Abstract:

**Comment #2**. Authors should describe more precisely what are the “adverse outcomes” they aim to study and what have they been focusing on.

**Response #2.** We have now described the adverse outcomes in the abstract and the revised study aim is provided in page 2, lines 14-17:

*“We aimed to investigate the risk of adverse pregnancy outcomes — preterm birth (PTB), spontaneous PTB, small for gestational age (SGA) birth, large for gestational age (LGA) birth, preeclampsia, and gestational diabetes mellitus (GDM) — by interpregnancy interval (IPI) for births following a previous miscarriage or induced abortion.”*

Introduction:

**Comment #3.** In the introduction, the authors should give more information why they choose to have pre-eclampsia and gestational diabetes in the study. It seems to be a kind of spin-off in the study, and no explanation why to study association of IPI with PE/GDM from the literature has been presented. The aims are a bit unclear and pre-eclampsia and GDM has not been mentioned as the aim. in fact, I would suggest to leave out PE and GDM form the study.

**Response #3.** Previous studies investigating the association between interpregnancy interval after a livebirth and preeclampsia and GDM have shown associations. However, there is a dearth of evidence exploring the association between these outcomes and interpregnancy intervals after miscarriage or induced abortion. We have included additional statement and citations supporting existing evidence.

Introduction page 5 lines 104-107.

*“Studies investigating risks of maternal complications after long IPI following a live birth indicated increased risks of gestational diabetes mellitus in (GDM) in Canada (OR 1.66, 95% CI: 1.39, 2.00) and preeclampsia in Australia (OR 1.28, 95% CI: 1.17, 1.41) [13, 14].*

Methods:

**Comment #4.1.** The major problem in the setting of this study is that the authors have included all the women during the study period, thus some women may appear in the data various times causing bias. The authors should reanalyze the study population by including each woman just once in the analyses.

**Response #4.1.** Apologies for the inadvertent error when we presented the number of women who contributed more than birth in the study population. We have carefully reviewed our data and corrected the numbers in which we had few observations of women contributing more than one birth during the study period (1,647 (3.4%)) women after induced abortion and 552 (2.3%) of women after induced abortion). To account the occurrence of multiple observations, we used cluster variance estimation. We have corrected our descriptions for the number of women that contributed more than one pregnancy during the study period. In addition, we have conducted additional sensitivity analysis restricting to births from women with only one miscarriage or induced abortion in the cohort. These analyses did not alter our results (Please see S9 & S10 Tables).

Methods section page 7 lines 154-156

*“A total of 1,647 (3.4%) women after miscarriages and 552 (2.3%) women after induced abortions contributed more than one birth in the study population”.*

To account the occurrence of more than one pregnancy from a woman, we considered robust cluster variance estimation methods in our model, and we have described this in the methods section in page 8, lines 175-177:

*“Robust cluster variance estimation was used to account for women who had more than one pregnancy during the study period.”*

We have also described our sensitivity analysis restricting births from women with only one miscarriage or induced abortion as follows in page 9, lines 197-198

*“We also conducted a sensitivity analysis restricting births from women with only one miscarriage (n=47,411) or induced abortion (n=23,185) in the cohort.”*

**Comment #4.2**. Also, the data concerning method of treatment (medical/surgical) as well as the duration of gestation in miscarriage/induced abortion would improve the study, as those factors are known to affect risks in pregnancy (for example multiple surgical abortions increase the risk of preterm birth).

**Response #4.2**. We agree with the reviewer that women having surgical terminations are prone to adverse pregnancy outcomes as opposed to medical abortion. Although we did not have information for the type of procedures used for pregnancy terminations, studies in Norway showed that pregnancy terminations were mostly conducted using medical termination methods and hence we anticipate that the contributions of surgical terminations were minimal. We have discussed this in the manuscript.

Discussion section page 19, lines 332-336.

“*Studies also indicated that uterine infections are mostly associated with surgical abortions than medical terminations. In Norway, medical abortions comprising more than 80% of all abortions conducted in 2013, and 95% of these terminations were performed in the first trimester [11]. Since 2009, medical abortion services were further expanded for women to get access to self-administered misoprostol to use at home for pregnancies terminations up to 12 weeks of gestation [12].”*

We agree with the reviewer that gestation age when the previous miscarriage or induced abortion occurred would influence our result. While the majority of miscarriages or induced abortions occurred in the first trimester, we did not have accurate information. We have now included the following statement in the limitation section.

Discussion section page 20 lines 355-358.

“*While gestational age when a previous miscarriage or an induced abortion occurred may be a potential confounder, we did not have such information for those miscarriages or induced abortions occurred within the first 12 weeks, which impacted our ability to consider this variable in the adjusted model.”*

Results:

**Comment #5.** As there are very few women in the IPI group > 24 months, I suggest to combine >24 months and > 60 months. Why not to have smoking in the model shown in the paper, if it does not change the results? Smoking is well known risk factor for SGA for example, and should be in the primary model.

**Response #5.** As suggested, we combined the last two categories and we have now run our models and updated the tables and text descriptions accordingly. Since we did not have smoking information for all study participants, we preferred to conduct a sensitivity analysis for those women with this information. See our description in the statistical analysis section page 9 lines 188-192.

*“We conducted sensitivity analyses additionally adjusting for pre‐pregnancy body mass index (BMI) (using restricted cubic splines placed at 5 knots placed at the 5^th^, 27.5^th^, 50^th^, 72.5^th^ and 95^th^ percentiles in the study population) and smoking during pregnancy (categorical: yes/no) for the index pregnancies with information available (n= 27,747 for births after a miscarriage; n =13,932 for births after an induced abortion).”*

Discussion:

**Comment #6.** Please see my previous comments on PE and GDM.

**Response #6.** See our response for **Comment #3**

**Reviewer #3**

**Comment #1.**  Delighted to review this manuscript that adds to the literature supporting early conception after miscarriage or induced abortion and calls into question WHO's guidance published in 2007.

**Response #1.** Thank you

**Comment #2.** Abstract: Please mention that only births following previous miscarriage/ induced abortion were looked at and not early pregnancy outcomes.

**Response #2.** We have revised abstract as suggested.

**Comment #3.1.** Author summary: "Available small studies exploring pregnancy outcomes after miscarriage (n<1,100) focused on the limited pregnancy outcomes such as recurrent miscarriage and preterm birth"- is this true? Some of the references cited have used larger datasets and the systematic review included many more pregnancies! This needs to be amended in the introduction section also.

**Response #3.1.** As the reviewer noted, we have discussed available large previous studies on the topic and then described the available studies with smaller sample size. We agree with the reviewer that without providing context, providing these statements describing studies with smaller sample in a bullet point in the “author summary” section could be confusing. We decided to remove this statement from this section. However, we have revised the related statement in the introduction section for more clarity and presented as follows:

Introduction section page 5 lines 109-111:

*“Besides the available large cohort studies described above, other studies with smaller sample size (n<1,100) were conducted to explore pregnancy outcomes after a miscarriage, however, these studies investigated limited outcomes such as recurrent miscarriage and preterm birth [15-18].*

**Comment #3.2.** I feel the biggest strength of this study is in being able to look at IPI of <3 months and study outcomes following both miscarriage and induced abortion in the same population. "Our study suggests that conceiving within 6 months after a miscarriage or an induced abortion is not associated with increased risks of adverse pregnancy outcomes" - this should be reported in "what did the researchers find?" Perhaps highlight that this was true for IPI less than 3 months too?

**Response 3.2.** As suggested, we have moved this bullet point further up in the section “what did the researchers find?". We have also indicated that these results were consistent for IPI <3 months. Please see the second bullet point under this section in the revised manuscript.

**Comment #4.1.** Methods: The ICD codes used to identify miscarriage include Hydatidiform mole - these have completely different management and reproductive trajectory and should not be included in miscarriage. In the big scheme of things the numbers are probably too few to make any difference but if possible, the analyses should be repeated excluding these cases.

**Response 4.1.** We acknowledge the reviewer for this insightful comment. We have now excluded molar pregnancies in our miscarriage definition. However, due to the small proportion of these observations (<0.2%), as the reviewer speculated, excluding these observations did not change our results. We have now excluded these cases from our miscarriage definition in our analysis cohorts and re-run our models.

Methods section page 6, lines 131-135.

*“From the patient registry, information on miscarriages before 12 completed gestational weeks were captured using the following ICD-10 codes: missed abortion (O02.1); other specified abnormal products of conception (O02.8); abnormal product of conception, unspecified (O02.9); spontaneous abortion (O03); and threatened abortion (O20.0).”*

**Comment 4.2.** Similarly, threatened abortion does not necessarily end in a miscarriage - in fact the vast majority of them don't. Not clear how more than one birth could have contributed to the index birth?

**Response #4.2.** In our study, threatened miscarriage and bleeding in pregnancy were considered as miscarriage if these pregnancies resulted in neither a registration of a livebirth nor a stillbirth in the birth registry database. We have clarified this in the revised manuscript. Please see the revised statements in page 7, lines 138-140 and presented as follows:

*“We only counted registrations of threatened abortion and bleeding in pregnancy codes as miscarriage or induced abortions if they did not subsequently end in a registration in the birth registry.”*

Comment 4.3: Statistical analysis is appropriate and described well but could perhaps benefit from justifying why log binomial regression was used and relative risks calculated as effect measure particularly since the covariates adjusted for were at the time of the index pregnancy.

**Response #4.3.** Please see our response to **Reviewer #1 in Comment #3**. Please also note that we conducted a sensitivity analysis adjusting for variables measured at the time of miscarriage or induced abortion (i.e., prior to interval).

**Comment #5.** Results: I would prefer to see tables 1 and 2 separately for miscarriage and induced abortion with the characteristics and outcomes presented by IPI. As such the reader gets no idea as to what number (%) of outcomes occurred in each IPI category. Although, these are presented in table 3 and so table 2 is perhaps redundant? Table 3 should mention which covariates were adjusted for to calculate ARR. were the same covariates adjusted for in all the models? What was the rationale behind including these?

**Response #5.** As suggested, we have now provided the results for births after a miscarriage and births after an induced abortion separately. See Tables 1-2 and S3 & S4 Tables in the revised version.

For some readers who might be interested to the proportion of outcomes of interest in the study cohort, we believe that presenting these results might be worthwhile. However, we decided to move these results to the supplementary information section. Please see S1 & S2 Tables.

For our adjusted analysis result presented in Table 3 and 4 in the revised version, we have provided footnotes for the adjusted variables. As we indicated in the methods section, we consistently adjusted for maternal age, gravidity, and year of birth for our models. We additionally adjust for smoking during pregnancy and pre pregnancy BMI in our sensitivity analysis. For all main and supplementary tables, we have indicated the adjusted variables as a footnote.

**Comment #6.1** Discussion: A main strength of this study is the ability to study separately the effects of increasing IPI after miscarriage and induced abortion in the same population. This inevitably begs the question as to how confident researchers were that there was no or minimal misclassification. Looking at the codes used to identify the cases I am not convinced that there was not some mixing. However, given that similar findings were observed in both groups, this probably does not matter. Another strength is the ability to study outcomes in IPI <3 months. This again begs the question as to how confident the researchers were that the two pregnancies were not the same, particularly as I note that threatened miscarriage and bleeding in pregnancy were included as miscarriage.

**Response #6.1.** We thank the reviewer for acknowledging the strengths of our research in its ability to separately investigating IPI after miscarriage and induced abortion in the same population. For ascertaining miscarriage and induced abortion, we followed the standard ICD 10 codes which allowed us to make distinction between these cases. There are clear clinical guidelines as to when the various ICD-10 codes should be used. To avoid confusion, we have now clarified our statements. Please see our response for **Comment #4.2** for threated abortion and bleeding in pregnancy.

**Comment 6.2.** A recent publication had suggested that even the current guidance of waiting for at least one normal period after a miscarriage was not justified. Would the authors like to comment on that vis a vis guidance in Norway? Lee Reicher, Ronni Gamzu, Yuval Fouks, Ofer Isakov, Yariv Yogev, Sharon Maslovitz,The effects of a postmiscarriage menstrual period prior to reconceiving, American Journal of Obstetrics and Gynecology, Volume 223, Issue 3,2020, Pages 444.e1-444.e5,ISSN 0002-9378,

<https://doi.org/10.1016/j.ajog.2020.06.051>.

**Response #6.2.** We thank the reviewer for the related publication based on a data from Israel. We have cited this paper in our study and discussed accordingly.

Discussion section page 17, lines 282-285.

*“Although not directly comparable to our study due to only including births after first trimester miscarriages and having a very small sample size (n=107), our results were consistent with a study from Israel that indicated that conception shortly after a spontaneous miscarriage was not associated with adverse maternal or neonatal outcomes [31].”*

**Comment #7.** I would urge the authors to proofread the manuscript carefully as there are several missed words and typographical errors throughout. Some references are also not formatted correctly (Se ref 11)

**Response #7.** We have now carefully reviewed the manuscript for any typographic error. We have also reviewed and formatted the references according to PLOS Medicine guidelines.

**Reviewer #4**

**Comment** Thank you for this well-written paper. I see this is a revision but cannot seem to access previous reviewer comments or responses. I have no further comments.

**Response:** Thank you. This version of the manuscript has only been previously reviewed by the editor of the journal who assessed the format of the manuscript. This is the first peer review report we have received for our submission.
