# Supplementary material for: Interpregnancy interval and adverse pregnancy outcomes among pregnancies following miscarriages or induced abortions in Norway (2008–2016): A cohort study
Source: PLoS Med. 2022 Nov 22;19(11):e1004129. doi: 10.1371/journal.pmed.1004129 (PMC9681073; doi:10.1371/journal.pmed.1004129)
Supplement: Attachment — Submitted filename: Point by point responses_R3.docx [file pmed.1004129.s014.docx]

**Point-by-point responses to the editors and reviewers**

**Requests from Editors:**

GENERAL

**Comment #1.** Thank you for reporting according to STROBE. Please add the following statement, or similar, to the Methods: "This study is reported as per the Strengthening the Reporting of Observational Studies in Epidemiology (STROBE) guideline (S1 Checklist)."

**Response #1.** We have provided the suggested text in the methods section at 7, lines 143-144.

*This study is reported as per the Strengthening the Reporting of Observational Studies in Epidemiology (STROBE) guideline [22] (S1 Checklist).*

ABSTRACT

**Comment #2.** Thank you for revising the abstract.

Thank you for including p-values. When reporting p-values please replace “p-value =…” with p = 0.07”. Please report p as <0.01 as opposed to p=0.00. Please check and amend throughout the abstract and main manuscript text Thank you for including limitations of your data set. Please also include limitations of the study methodology (i.e. retrospective, observational etc), any problems with the use of registry data (reliability)?

**Response #2.** As suggested, we have revised p-values throughout the manuscript. We have also revised the limitation section describing the inherent caveats of retrospective registry linkage studies on page 3, lines 40-43:

*Inherent to retrospective registry-based studies, we did not have information on potential confounders such as pregnancy intention and health seeking bahaviour. Furthermore, we only had information on miscarriages which resulted in contact with the health-care system.*

AUTHOR SUMMARY

**Comment #3.** Thank you for revising the author summary I suggest “review of current international….” instead of “revision”

**Response #3.** Thank you. We have corrected the text as suggested.

METHODS and RESULTS

**Comment #4.** Table 1: Thank you making revisions. I suggest a few further thing. There seems to be a redundant third unfilled column on the right please remove this. Suggest relabeling the heading to column 2: “Number of participants” or something similar. Perhaps also include the total number of ppts in the table in an appropriate place.

**Response #4.** Thank you for noting this. We have now removed the redundant column.

DISCUSSION

**Comment #5.** Please remove sub headings and include the strengths and limitations section within the discussion which should be structured as follows: a short, clear summary of the article's findings; what the study adds to existing research and where and why the results may differ from previous research; strengths and limitations of the study; implications and next steps for research, clinical practice, and/or public policy; one-paragraph conclusion.

**Response #5.** We have removed all sub-headings and incorporated the specified elements into the discussion.

**Comment #6.** Please remove the funding statement and COI from the end of the manuscript and include only in the submission form

**Response #6.** We have removed the funding statement and COI from the main manuscript

REFERENCES

**Comment #7.** Please see our website for reference guidelines <https://aus01.safelinks.protection.outlook.com/?url=https%3A%2F%2Fjournals.plos.org%2Fplosmedicine%2Fs%2Fsubmission-guidelines%23loc-references&amp;data=05%7C01%7Cgizachew.tessema%40curtin.edu.au%7C4dd8e76a8a754b13136908daae0380b6%7C5a740cd757684d09ae13f706b09fa22c%7C0%7C0%7C638013627314116313%7CUnknown%7CTWFpbGZsb3d8eyJWIjoiMC4wLjAwMDAiLCJQIjoiV2luMzIiLCJBTiI6Ik1haWwiLCJXVCI6Mn0%3D%7C3000%7C%7C%7C&amp;sdata=JGNlL9PI6a1AfnvqQ8XxvOWlKjQQf1VYWaaDTLr%2Fcpg%3D&amp;reserved=0>

Please ensure journal name abbreviations are those found in the National Center for Biotechnology Information (NCBI) databases.

**Response #7.** We have checked and corrected all the references according to the journal’s guidelines.

**Comment #8.** To help us extend the reach of your research, please provide any Twitter handle(s) that would be appropriate to tag, including your own, your coauthors’, your institution, funder, or lab. Please respond to this email with any handles you wish to be included when we tweet this paper.

**Response #9.** As requested, we have now included relevant twitter handles of the co-authors and funders and our institutions:
@Giz_tessema; @GavinFPereira; @AnnetteKRegan; @Jendunneq; @CurtinUni @CurtinMedia; @RaineFoundation; @nhmrc; @Folkehelseinst; @theCEFH.

**Comments from the academic editor:**

**Comment #9.** I have one remaining thing that I think they need to clarify. Their response to comment 4.2 in relation to gestational age (page 11) is, for me, inadequate. They refer to the gestational age of the miscarriage or induced abortion as a "confounder". But it is not. The gestational age at the time of the miscarriage or induced abortion is a determinant of the exposure. These events are broadly classified into first trimester and second trimester. The vast majority of both are first trimester. It seems plausible to me that later miscarriage or induced abortion (e.g. ~20 weeks) might be more like a previous birth where a very short IPI is thought to be associated with greater risks. I think that they need to spell out in the discussion that they were unable to sub-classify miscarriages or induced abortions by gestational age, that the vast majority will be first trimester and their results should be extrapolated cautiously to second trimester miscarriages or induced abortions.

**Response #9.** We thank the editor for this additional comment and suggestion. We agree with the editor that late miscarriages or induced abortions might be more like a previous birth with a potential of increasing risk if followed by a short interval. We have clarified in the revised text that although we did not have information on gestational age for miscarriages or induced abortions occurred within the first trimester, we did know whether the miscarriage or induced abortion occurred prior to or after 12 weeks of gestation. We believe that this helped to minimise the risk of misclassification. We have now revised our discussion in page 20, lines 375-380 and provided as follows:

*“Although the gestational age at previous miscarriage or induced abortion affects the IPI , we did not have information on exact gestational age for those miscarriages or induced abortions occurred before 12 weeks, which comprised 99% of miscarriages and 96% of induced abortions. However, we did have information on gestational age at miscarriages or induced abortions after 12 weeks and hence the risk of misclassification of exposure is minimal for these pregnancies.”*

**Comments from Reviewers:**

Reviewer #1: The authors have addressed my concerns and I now recommend publication

**Response:** Thank you also for the time and constructive comments

Reviewer #3: Many thanks for taking on board my comments and suggestions and revising the manuscript accordingly. I have no further comments and feel that this paper will add to the body of evidence supporting early conception following miscarriage and induced abortion.

**Response:** We also thank you for the time and constructive comments that improved the quality of our manuscript.
